# BERT-Kgly: A Bidirectional Encoder Representations From Transformers (BERT)-Based Model for Predicting Lysine Glycation Site for *Homo sapiens*

**DOI:** 10.3389/fbinf.2022.834153

**Published:** 2022-02-18

**Authors:** Yinbo Liu, Yufeng Liu, Gang-Ao Wang, Yinchu Cheng, Shoudong Bi, Xiaolei Zhu

**Affiliations:** School of Sciences, Anhui Agricultural University, Hefei, China

**Keywords:** protein lysine glycation, BERT, biological sequence, natural language processing, posttranslational modification (PTM), embedding

## Abstract

As one of the most important posttranslational modifications (PTMs), protein lysine glycation changes the characteristics of the proteins and leads to the dysfunction of the proteins, which may cause diseases. Accurately detecting the glycation sites is of great benefit for understanding the biological function and potential mechanism of glycation in the treatment of diseases. However, experimental methods are expensive and time-consuming for lysine glycation site identification. Instead, computational methods, with their higher efficiency and lower cost, could be an important supplement to the experimental methods. In this study, we proposed a novel predictor, BERT-Kgly, for protein lysine glycation site prediction, which was developed by extracting embedding features of protein segments from pretrained Bidirectional Encoder Representations from Transformers (BERT) models. Three pretrained BERT models were explored to get the embeddings with optimal representability, and three downstream deep networks were employed to build our models. Our results showed that the model based on embeddings extracted from the BERT model pretrained on 556,603 protein sequences of UniProt outperforms other models. In addition, an independent test set was used to evaluate and compare our model with other existing methods, which indicated that our model was superior to other existing models.

## 1 Introduction

As one of the most important posttranslational modifications (PTMs) of proteins, glycation is a two-step non-enzymatic reaction that is different from glycosylation, which is an enzyme-dependent reaction (Stitt 2001). Advanced glycation end products (AGEs) generated in the reaction are involved in different human diseases (Vlassara et al., 1994; Ling et al., 1998; Ahmed et al., 2005), such as diabetes, Alzheimer’s disease, and Parkinson’s disease. The identification of glycation sites in proteins would be of great benefit for the understanding of the biological function of protein glycation and treatment of the related diseases. In addition to human metabolism, protein glycation is also an unavoidable part of plant metabolism and proteotoxicity (Rabbani et al., 2020).

Different methods have been developed for detecting lysine glycation (Kgly) sites. Wet experiment methods such as mass spectrometry (Thornalley et al., 2003) and electrochemical chip (Khan and Park 2020) have been used to identify lysine glycation sites. However, wet experiment methods are both cost- and time-consuming. Alternatively, several in silico methods (Johansen et al., 2006; Liu et al., 2015; Ju et al., 2017; Xu H. et al., 2017; Zhao et al., 2017; Islam et al., 2018; Chen K. et al., 2019; Yu et al., 2019; Khanum et al., 2020; Yang et al., 2021; Yao et al., 2021) have been developed to predict the Kgly sites efficiently. In a pioneer work, Johansen et al. proposed a predictor, GlyNN, built by neural networks based on a dataset with 89 Kgly sites and 126 non-Kgly sites of 20 proteins (Johansen et al., 2006). Later, Liu et al. developed a model, PreGly, by using support vector machine (SVM) for detecting Kgly sites (Liu et al., 2015). They used the same dataset as Johansen et al.’s study and generated three kinds of sequence features that were selected by using the maximum relevance minimum redundancy (mRMR) and the incremental feature selection (IFS) methods. Based on the larger training dataset, Xu et al. built a Kgly site prediction model, Gly-PseAAC, based on sequence order information and position-specific amino acid propensity (Xu Y. et al., 2017). By using the same dataset as Xu et al.’s study, Ju et al. constructed a model, BPB_GlySite, to predict glycation sites by using a single feature of bi-profile Bayes (BPB) (Ju et al., 2017). By using Xu et al.’s dataset as a training dataset, Zhao et al. built a model, Glypre, based on fused multiple features *via* using a two-step feature selection method (Zhao et al., 2017). In addition, they used another two datasets to test the generalization of their model. Benchmarked on Xu et al.’s dataset and other two datasets, Islam et al. proposed a method, iProtGly-SS, to predict Kgly by searching the optimal feature subset from sequential features, physicochemical properties, and structural features using an incremental group-based feature selection algorithm (Islam et al., 2018). Based on predicted structural properties of residues, Reddy et al. developed a model, GlyStruct, based on the SVM (Reddy et al., 2019). Leveraging Xu et al.’s dataset as training dataset, Yao et al. developed a model, ABC-Gly, by selecting the optimal feature subset with a two-step feature selection method by combining the Fisher score and an improved binary artificial bee colony algorithm (Yao et al., 2021). All the previous methods were built on the dataset with less than 500 Kgly sites; however, four other methods, PredGly (Yu et al., 2019), Gly-LysPred (Khanum et al., 2020), MUscADEL (Chen Z et al., 2019), and MultiLyGAN (Yang et al., 2021), which were built on datasets with more than 1,000 Kgly sites. For building PredGly, Yu et al. (2019) collected Kgly sites from PLMD (Xu H. et al., 2017) and used CD-HIT (Huang et al., 2010) to remove the redundancy for protein sequences and peptide segments, with a cutoff of 30%. The dataset contains 3,969 non-redundant Kgly sites and 82,270 non-Kgly sites. Based on the dataset, they built their model by selecting an optimal feature subset via XGBoost (Chen and Guestrin 2016). By collecting Kgly sites from UniProt (https://www.uniprot.org/), Khanum et al. obtained their dataset with 1,287 Kgly sites and 1,300 non-Kgly sites by using CD-HIT to remove the redundancy, with a cutoff of 60%, and then built their model by using random forest (Khanum et al., 2020). Both MUscADEL (Chen K et al., 2019) and MultiLyGAN (Yang et al., 2021) were developed to predict multiple lysine modification sites. For MUscADEL, Chen et al. collected Kgly sites for both *Homo sapiens* and *Mus musculus* from the PhosphoSitePlus database (Hornbeck et al., 2015), and then removed the redundancy of protein sequences by using CD-HIT (Huang et al., 2010), with a cutoff of 30%. Based on the dataset with 3,209 Kgly sites, they built their model by using a deep learning algorithm. In MultiLyGAN (Yang et al., 2021), Yang et al. collected lysine modification sites from the CPLM2.0 database (Liu et al., 2014), and after removing redundancy by using CD-HIT at the segment level with a cutoff of 40%, they obtained 1,454 Kgly sites. Their model is a multiple-label model built with data augmentation by conditional Wasserstein generative adversarial networks. The details of all these tools are summarized in Supplementary Table S1.

Although considerable progress has been made for differentiating Kgly sites and non-Kgly sites, the performance of these methods is still not satisfactory. One possible reason is the limitation of the representability of the features used before. The powerful representability of the Bidirectional Encoder Representations from Transformers (BERT) (Devlin et al., 2019) model has been demonstrated in the current field of natural language processing (NLP). By considering the biological sequences as sentences, their representability has been explored in a variety of works. Rives et al. pretrained protein language BERT models based on 250 million protein sequences (Rives et al., 2021) and explored the representations of these models, and their results demonstrated that the information of protein structure and function was encoded in representations of these models. Rao et al. pretrained protein language BERT models based on 31 million protein sequences (Rao et al., 2019). Zhang et al. pretrained protein language BERT models based on 556,603 protein sequences (Zhang et al., 2021). The embeddings extracted by the BERT pretrained models have been used as features for classification in bioinformatics. With the embeddings, Le et al. have developed a model to predict enhancers (Le et al., 2021), Qiao et al. have developed a model to predict Kcr sites (Qiao et al., 2021). Thus, the embeddings of pretrained BERT models may be helpful for building a more effective model for Kgly sites prediction.

**FIGURE 1:**
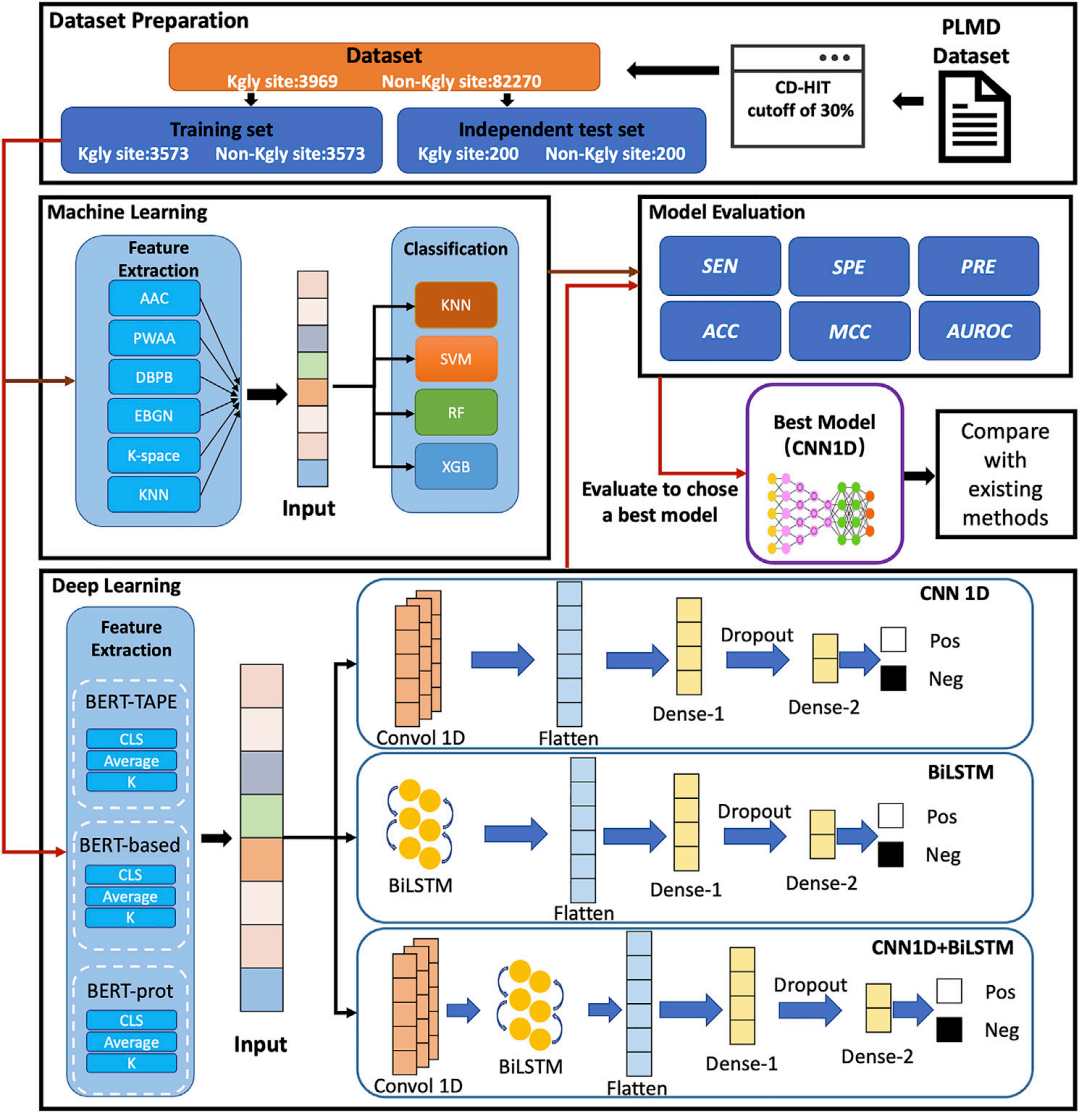
Flowchart for building our model.

In this study, we proposed a computational approach called BERT-Kgly to improve the predictive performance of lysine glycation sites. Considering peptide segments as sentences, the embeddings were extracted from three different pretrained BERT models which were fed to the downstream classifiers for Kgly site prediction. In addition, several traditional features were also extracted, and their performance was compared with the embeddings of BERT. Furthermore, the built model with embeddings of BERT was compared with the existing methods using an independent test set. Empirical studies showed that our model, BERT-Kgly, outperforms other methods, with an area under the receiver operating characteristic curve (AUROC) of 0.69. The workflow of BERT-Kgly is shown in Figure 1.

## 2 Methods and Materials

### 2.1 Data Sets

In this study, we used the same dataset as that collected by Yu et al. (Yu et al., 2019), for their dataset is the largest for *Homo sapiens*, as shown in Supplementary Table S1. The dataset was collected from the PLMD database (http://plmd. biocuckoo. org/) (Xu Y. et al., 2017). The redundancy of the dataset was removed by a two-step process on the protein level and segment level with CD-HIT (Huang et al., 2010) by using a cutoff of 30%, respectively. Overall, the dataset contains 3,969 positive and 82,270 negative samples, and about 90% of positive samples 3,573) and an equal number of negative samples were selected randomly for training. For the independent test set, Yu et al. selected 200 positive and 200 negative samples from the remaining datasets. In Yu et al.’s dataset, each sample contains 31 residues with the lysine in the middle. The protein segments with different lengths can be used to build our model. In previous works, (Yu et al., 2019 and Zhao et al., 2017). have demonstrated that the segments with 15 downstream and upstream residues showed the best performance. All data and codes are available at https://github.com/yinboliu-git/Gly-ML-BERT-DL.

### 2.2 Feature Extraction

#### 2.2.1 Embeddings of BERT Pretrained Models

We used three different BERT pretrained models to encode the peptide segments in our datasets, which are the initial natural language BERT-Base model released by Google Research (Devlin et al., 2019), Zhang et al.’s BERT model (Zhang et al., 2021) which was pretrained on 556,603 protein sequences from UniProt (named as BERT-prot), and the TAPE model (Rao et al., 2019) which is based on 31 million protein domains from Pfam. These models encode a 768-dimensional vector corresponding to each residue of the peptide segments.

The Bidirectional Encoder Representations from Transformers (BERT) model was developed by Devlin et al. (Devlin et al., 2019), which has achieved new state-of-the-art results on 11 natural language processing (NLP) tasks. The architecture of BERT is a multilayered bidirectional Transformer encoder, which jointly conditions on both left and right context using the attention mechanism in all encoder layers and processes all words in the sentence in parallel. The network structures of all the encoder layers are the same, which mainly consisted of two sublayers: the multi-head self-attention layer and the feed-forward neural network layer. In addition, a residual connection is added on each of the sublayer; thus, the output of each sublayer is LayerNorm (x + Sublayer(x)). When a sentence is inputted into the BERT model, each word was encoded by three embeddings: token embeddings, segment embeddings, and position embeddings. Then, we can obtain context-dependent features from different encoder layers of the model.

For comparison, we also calculated six types of traditional sequence-based features as follows.

#### 2.2.2 Amino Acid Composition

As a classic sequence coding feature, amino acid composition (AAC) has been used extensively for PTM sites prediction (Xu et al., 2016; Yu et al., 2019; Zhang et al., 2019; Basith et al., 2021). It counts the occurrence frequency of each of the 20 natural amino acids and one complementary amino acid “O” in the peptide segments.

#### 2.2.3 K-Spaced Amino Acid Pair Composition

K-spaced amino acid pair composition or composition of k-spaced amino acid pairs (CKSAAP) is another sequence encoding scheme that has been employed to predict various PTMs (Chen et al., 2008; Fu et al., 2019; Wu et al., 2019; Lv et al., 2020; Chen et al., 2021). This method mainly calculates the frequency of different pairs of amino acids separated by k-length peptides. If we used $A_{1}X\left\{k\right\}A_{2}$ to represent k-spaced amino acid pairs, both $A_{1}$ and $A_{2}$ can be the 21 types of amino acids, so there are 441 types of k-spaced amino acid pairs. Each of them can be calculated as follows:

$$f\left(A_{1}X\left\{k\right\}A_{2}\right)=N\left(A_{1}X\left\{k\right\}A_{2}\right)/\left(L-k+1\right),$$

where L represents the length of the segment and $N\left(A_{1}X\left\{k\right\}A_{2}\right)$ is the occurrence frequency of $A_{1}X\left\{k\right\}A_{2}.$

#### 2.2.4 Position Weight Amino Acid Composition

Position weight amino acid composition (PWAA), which is first proposed by Shi et al. (Shi et al., 2012), is used to extract the sequence order information of amino acid residues around target residues. For each of the 20 types of residues, the feature can be calculated by using the following equation:

$$\mathrm{PWAA}\left(i\right)=\;\frac{1}{L\left(L+1\right)}\overset{L}{\underset{j=-L}{\sum }}x_{i,j}\left(j+\frac{\left|j\right|}{L}\right),$$

where i denotes one of the 20 types of residues, L represents the number of upstream or downstream residues, and $x_{i,j}$ describes if the type of the residue on position j of the peptide segment is the same as i , if true then its value is 1, otherwise 0.

#### 2.2.5 Dipeptide Bi-Profile Bayes (DBPB)

The bi-profile Bayes feature proposed by Shao et al. (Shao et al., 2009) is used to represent the occurrence probability of each type of residues on each position of the positive peptide segments and negative peptide segments, respectively. Thus, the dipeptide bi-profile Bayes (DBPB) feature is used to represent the occurrence probability of each type of dipeptides on each position of the positive peptide segments and negative peptide segments, respectively. These probabilities were first calculated based on the data used for training which were then assigned to the peptide used for testing. Note that the data used for validation could not be used for calculating the probabilities in the cross-validation stage.

#### 2.2.6 Encoding Based on Grouped Weight (EBGW)

For calculating this kind of feature, the 20 types of residues were first classified into four different groups according to the charge and hydrophobicity properties. Then, the four groups were further divided into three categories. For each category, the 20 types of residues were divided into two classes, so that a binary representation can be obtained for a residue according to which class it belongs to. Thus, a peptide segment with length L can be represented as a binary vector with the same length. Totally, we obtained three binary vectors for each peptide segment. Each vector was then divided into J sub-vectors increasing in length, the feature for each sub-vector were calculated as follows: X(j) = sum (sub-vector(j))/length (sub-vector(j)). In all, we obtained 3 J-dimension feature vectors for each peptide segment. J was set as 5 according to previous studies (Shi et al., 2012; Yu et al., 2019).

#### 2.2.7 K-Nearest Neighbor (KNN) Feature

The k-nearest neighbor feature counts the positive samples percentage of the k nearest samples in the training dataset to the query sample. For peptide segment samples, the distance between two different samples is represented by sequence similarity which is calculated as follows:

$$\mathrm{Dist}\left(S_{1},S_{2}\right)=1-\frac{\sum_{i=1}^{L}\mathrm{Sim}\left(S_{1}\left(i\right),S_{2}\left(i\right)\right)}{L},$$

where L is the length of the peptide segments and $S_{1}\left(i\right)\;and\;S_{2}\left(i\right)$ represent the *i*th residues of the two segments $S_{1}$ and $S_{2}$ , respectively. The similarity between $S_{1}\left(i\right)$ and $S_{2}\left(i\right)$ is computed as follows:

$$\mathrm{Sim}\left(m,n\right)=\frac{B\left(m,n\right)-\min\left(B\right)}{\max\left(B\right)-\min\left(B\right)},$$

where B represents the BLOSUM62 substitution matrix (Henikoff and Henikoff 1992) and max(B) and min(B) represent the largest and smallest values of the matrix, respectively. Given k = 2,4,8,16,32, we generated 5D feature vectors for a given peptide segment.

### 2.3 Machine Learning and Deep Learning Algorithms

#### 2.3.1 SVM

A support vector machine (SVM) is one of the most popular learning algorithms which has been used extensively in bioinformatics (Chen Z. et al., 2019; Zhu et al., 2019; Chen et al., 2020). SVM was first proposed by Vapnik (1995), the main idea of which is to determine a hyperplane to maximize the margin between different classes. In this study, the sklearn package for Python 3 (https://www.python.org) was used to build the SVM classifiers.

#### 2.3.2 Random Forest

Random forest (RF) (Breiman 2001) is an ensemble learning algorithm by using a decision tree as a base learner. Based on different training sets which were sampled from the original training dataset and different feature subsets which were randomly selected from the original feature set, multiple decision trees were built. The class of a test sample is determined based on the voting result of all the base decision trees. In this work, the sklearn package for Python 3 (https://www.python.org) was used to build the RF classifier.

#### 2.3.3 XGBoost

XGBoost (Chen and Guestrin 2016) is an also ensemble learning algorithm using a decision tree as a base learner. Based on the gradient boosting decision tree (GBDT) (Friedman 2001), the regularization term was added which effectively prevents the problem of overfitting. The algorithm not only inherits the good performance of the original boosting algorithm but also shows the advantage to process sparse data and high dimensional data. In this study, the xgboost package for Python 3 is used to build the XGBoost classifier.

#### 2.3.4 KNN

The K-nearest neighbor classification rule was first proposed by Cover et al. (Cover and Hart 1967), in which the new sample was classified based on its nearest set of previously classified samples. The algorithm does not depend on any special distribution of the samples, which has been a ubiquitous classification tool with good scalability.

#### 2.3.5 CNN

As a famous deep network, the convolutional neural network (CNN) (Krizhevsky et al., 2012), was originally used in the field of computer vision which has been used extensively in many other fields. CNN is composed of a convolutional layer and a pooling layer. In this study, our network includes an input layer, a 1-dimensional convolutional layer with 64 filters, a flatten layer, a dropout layer, a dense layer with 32 nodes, and an output layer. The Adam algorithm was selected as the optimizer, and the cross-entropy loss formula was selected as the loss function.

#### 2.3.6 BiLSTM

The long short-term memory (LSTM) network (Hochreiter and Schmidhuber 1997) is a variant of a recurrent neural network (Schuster and Paliwal 1997). By combining forward LSTM and backward LSTM, a bidirectional long short-term memory (BiLSTM) network (Zhang et al., 2015) was proposed to model the context information and effectively capture bidirectional semantic dependencies in natural language processing (NLP). In this study, the architecture of our network is composed of an input layer, a BiLSTM layer with 128 hidden units, a flatten layer, a dense layer with 32 nodes, a dropout layer, and an output layer. The Adam algorithm was selected as the optimizer, and the cross-entropy loss formula was selected as the loss function.

#### 2.3.7 CNN + BiLSTM

In addition, we also designed a network that combined CNN and BiLSTM. Specifically, the network contains an input layer, a 1D CNN layer with 64 filters, a BiLSM layer wit 128 hidden units, a flatten layer, a dense layer of 32 nodes, a dropout layer, and an output layer. The Adam algorithm was selected as the optimizer, and the cross-entropy loss formula was selected as the loss function.

### 2.4 Model Evaluation Parameters

Generally, we used the area under the receiver operating characteristic (ROC) curve as our main metric to evaluate the models. The ROC curve can evaluate the prediction performance of the proposed method in the whole decision value range, and the area under the curve (AUROC) is often used to quantify the performance of the model. In addition, we also calculated 5 other metrics which are sensitivity (SEN), specificity (SPE), precision (PRE), accuracy (ACC), and Matthews correlation coefficient (MCC). The five metrics are defined as follows:

$$\mathrm{SEN}=\;\frac{\mathrm{TP}}{\mathrm{TP}+\mathrm{FN}}\;,$$

$$\mathrm{SPE}=\frac{\mathrm{TN}}{\mathrm{TN}+\mathrm{FP}}\;,$$

$$\mathrm{PRE}=\frac{\mathrm{TP}}{\mathrm{TP}+\mathrm{FP}}\;,$$

$$\mathrm{ACC}=\frac{\mathrm{TP}+\mathrm{TN}}{\mathrm{TP}+\mathrm{FP}+\mathrm{TN}+\mathrm{FN}}\;,\text{ and}$$

$$\mathrm{MCC}=\frac{\mathrm{TP*TN}-\mathrm{FP*FN}}{\sqrt{\left(\mathrm{TP}+\mathrm{FP}\right)*\left(\mathrm{TN}+\mathrm{FN}\right)*\left(\mathrm{TN}+\mathrm{FP}\right)*\left(\mathrm{TN}+\mathrm{FN}\right)}}\text{ ,}$$

where TP (true positive) means the number of predicted Kgly sites are actual Kgly sites, FP (false positive) means the number of predicted Kgly sites are actual non-Kgly sites, TN (true negative) means the number of predicted non-Kgly sites are actual non-Kgly sites, and FN (false negative) means the predicted non-Kgly sites are actual Kgly sites.

## 3 Results

### 3.1 Sequence Discrepancy Between Positive and Negative Samples in the Benchmark Dataset

**FIGURE 2:**
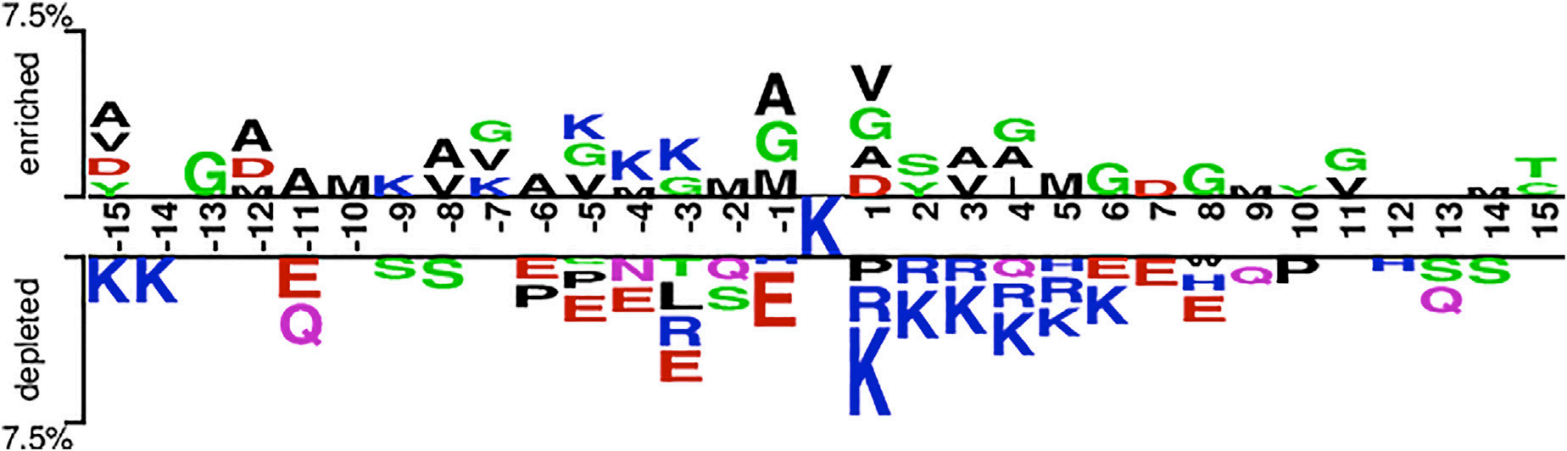
Overall sequence pattern discrepancy between positive and native samples illustrated by Two Sample Logo.

Based on the hypothesis that the sequence patterns of positive samples are different from that of the negative sample, we are able to develop machine learning methods to discriminate Kgly sites from non-Kgly sites. The overall pattern discrepancy could be visualized by Two Sample Logo (Vacic et al., 2006). The distribution and preference of the flanking residues of the central lysine were analyzed. Figure 2 shows that amino acids G, V, M, and A are enriched in positive samples, which are all uncharged residues. On the contrary, the amino acids K, R, and E are depleted in negative samples, which are all charged residues. In addition, most of the depleted amino acids E of the negative samples are on the left of central lysine sites at positions −11, −6, −5, −4, −3, and −1. On the other hand, residues R and K of the negative samples are depleted on the right of central lysine sites at positions +1, +2, +3, +4, and +5. Although there is a difference in the distribution and preference between positive and negative samples, the overall enriched or depleted ratio for a specific sequential position is less than 7.5%. Thus, the context information may be helpful to build a classification model.

### 3.2 Model Performance Based on Embeddings of Different Pretrained BERT Models

#### 3.2.1 Model Performance Based on the Embedding of Token “CLS”

From pretrained BERT models, the token “CLS” is often used for downstream classification tasks, so we extracted the embeddings of the token “CLS” of different segments to build our models. Three deep networks were used to build our models including 1D CNN, BiLSTM, and 1D CNN + BiLSTM. The grid search has been used to optimize the hyperparameters such as batch_size, learning rate, and epochs, for which the ranges are shown in Supplementary Table S2. Thus, we obtained the optimal models for different networks and different embeddings (Supplementary Tables S3, S4, S5). Table 1 shows that the performance of the models based on the embeddings extracted from BERT-prot is generally better than the embeddings extracted from BERT-base and TAPE according to values of AUROC. The ROC curves can be found in the supplemental materials (Supplementary Figure S1). In addition, the performance of the models based on the embeddings extracted from BERT-Base is better than that of TAPE. Note that BERT-Base is a pretrained natural language model, BERT-prot is a pretrained protein language model based on about 560,000 sequences, and TAPE is a pretrained protein language model based on about 31 million sequences.

**TABLE 1:** Cross-validation performance of models based on embeddings of token “CLS” extracted from different pretrained BERT models.

	Deep networks	Sen	Spe	Pre	MCC	ACC	AUROC
BERT-Base	1D CNN	**0.668**	0.440	0.548	0.117	0.554	0.581
	BiLSTM	0.602	0.485	0.547	0.096	0.544	0.571
	1D CNN + BiLSTM	0.640	0.454	0.544	0.105	0.547	0.572
BERT-prot	1D CNN	0.569	0.616	0.603	**0.191**	**0.592**	**0.643**
	BiLSTM	0.604	0.576	0.594	0.188	0.590	0.638
	1D CNN + BiLSTM	0.588	0.592	**0.604**	**0.191**	0.590	0.639
BERT-TAPE	1D CNN	0.308	**0.685**	0.505	-0.007	0.497	0.485
	BiLSTM	0.469	0.541	0.507	0.012	0.505	0.505
	1D CNN + BiLSTM	0.409	0.588	0.498	-0.003	0.498	0.498

Bold values means the highest values of that column in the tables.

Furthermore, based on the embeddings of BERT-Base and BERT-prot, Table 1 also shows that the models with 1D CNN outperform the models with BiLSTM and 1D CNN + BiLSTM. But for the embeddings of TAPE, the model with BiLSTM outperforms the other two networks. Overall, with the 1D CNN network, the model based on the embeddings of BERT-prot achieved the best performance.

#### 3.2.2 Model Performance Based on the Embedding of Token “K”

In this work, the middle residue of all the peptide segments is K (lysine), so we explored if we could use the embeddings of the middle Ks to build our models. Table 2 shows that the performance of the models based on the embeddings extracted from BERT-Base and BERT-prot is similar according to the values of AUROC, and the performance of the model based on the embeddings extracted from TAPE is inferior to that of the other two. Moreover, based on the embeddings of BERT-Base and BERT-prot, Table 2 shows that the models with 1D CNN again outperform the models with BiLSTM and 1D CNN + BiLSTM. But for the embeddings of TAPE, the model with BiLSTM outperforms the other two networks. Overall, with the 1D CNN network, the model based on the embeddings of BERT-Base achieved the best performance.

**TABLE 2:** Cross-validation performance of models based on embeddings of the central “K” extracted from different pretrained BERT models.

	Deep networks	Sen	Spe	Pre	MCC	ACC	AUROC
BERT-base	1D CNN	**0.698**	0.464	0.580	0.185	0.581	**0.634**
	BiLSTM	0.587	0.596	**0.600**	0.188	0.591	0.626
	1D CNN + BiLSTM	0.551	**0.616**	0.599	0.174	0.584	0.628
BERT-prot	1D CNN	0.610	0.580	0.593	**0.191**	**0.595**	0.632
	BiLSTM	0.583	0.575	0.586	0.164	0.579	0.618
	1D CNN + BiLSTM	0.682	0.483	0.573	0.174	0.582	0.630
BERT-TAPE	1D CNN	0.536	0.478	0.505	0.013	0.507	0.509
	BiLSTM	0.510	0.530	0.521	0.040	0.520	0.517
	1D CNN + BiLSTM	0.473	0.549	0.512	0.022	0.511	0.514

Bold values means the highest values of that column in the tables.

#### 3.2.3 Model Performance Based on the Average Embeddings of the Peptide Segment

The average embedding of the tokens of the whole sentence can also be used for downstream classification tasks. In this study, the average embedding of the 31 tokens in a peptide segment was extracted to build our models. Table 3 shows that the whole profile of the results is similar to that of the results shown in Table 2. The model based on the combination of BERT-prot and 1D CNN network achieved the best performance.

**TABLE 3:** Cross-validation performance of models based on embeddings of the central “average” extracted from different pretrained BERT models.

	Deep networks	Sen	Spe	Pre	MCC	ACC	AUROC
BERT-base	1D CNN	0.526	0.605	0.589	0.144	0.566	0.606
	BiLSTM	0.699	0.423	0.556	0.144	0.561	0.600
	1D CNN + BiLSTM	0.610	0.510	0.568	0.138	0.560	0.597
BERT-prot	1D CNN	0.595	0.595	**0.598**	0.192	0.595	**0.640**
	BiLSTM	0.613	0.584	0.598	**0.199**	**0.599**	0.636
	1D CNN + BiLSTM	**0.703**	0.465	0.583	0.194	0.584	0.639
BERT-TAPE	1D CNN	0.517	0.491	0.501	0.008	0.504	0.503
	BiLSTM	0.476	0.514	0.491	-0.008	0.495	0.496
	1D CNN + BiLSTM	0.369	**0.633**	0.540	-0.026	0.501	0.501

Bold values means the highest values of that column in the tables.

All in all, three different types of embeddings extracted from three different pretrained models were fed to three different deep networks. It turned out that the representability of the embeddings extracted from BERT-prot is better than that of BERT-base and TAPE in this study. Moreover, the model based on 1D CNN shows the best performance.

### 3.3 Model Performance Based on Handcrafted Feature With Machine Learning Algorithms

To demonstrate the effectiveness of the embeddings of pretrained models, we also calculated six kinds of handcrafted features (HCFs) which were then used to build models based on four machine learning algorithms, namely, XGBoost, random forest, SVM, and KNN. The hyperparameters of these algorithms were also optimized (Supplementary Table S6,S7). The performance of these models is shown in Table 4. Overall, the models based on AAC and CKSAAP were superior to the models based on the other four kinds of features according to the values of AUROCs. The ROC curves can be found in supplemental materials (Supplementary Figure S2). For all six kinds of features, the models based on XGBoost show the best or near-best performance. The best model was obtained by combining AAC and XGBoost, for which the AUROC is 0.633.

**TABLE 4:** Cross-validation performance of models based on handcrafted features.

HCF	Classifier	Sen	Spe	Pre	MCC	ACC	AUROC
AAC	KNN	0.587	0.514	0.547	0.102	0.551	0.571
	RF	0.664	0.526	0.584	**0.192**	0.595	0.631
	SVM	0.530	0.586	0.562	0.116	0.558	0.590
	XGBoost	0.638	0.551	0.587	0.191	**0.595**	**0.633**
DBPB	KNN	0.529	0.528	0.529	0.057	0.528	0.537
	RF	0.551	0.537	0.544	0.088	0.544	0.558
	SVM	0.522	0.575	0.551	0.097	0.548	0.570
	XGBoost	0.547	0.545	0.546	0.092	0.546	0.567
EBGW	KNN	0.537	0.498	0.517	0.035	0.517	0.527
	RF	0.707	0.386	0.535	0.099	0.547	0.564
	SVM	0.565	0.535	0.549	0.100	0.550	0.569
	XGBoost	0.688	0.413	0.540	0.106	0.551	0.568
KNN	KNN	0.418	0.617	0.522	0.036	0.518	0.523
	RF	**0.710**	0.368	0.529	0.084	0.539	0.555
	SVM	0.566	0.510	0.537	0.077	0.538	0.555
	XGBoost	0.685	0.396	0.531	0.085	0.541	0.554
CKSAAP	KNN	0.591	0.505	0.544	0.096	0.548	0.566
	RF	0.639	0.533	0.578	0.173	0.586	0.626
	SVM	0.554	0.625	**0.596**	0.180	0.590	0.629
	XGBoost	0.607	0.568	0.585	0.176	0.588	0.629
PWAA	KNN	0.517	0.517	0.517	0.033	0.517	0.527
	RF	0.483	0.629	0.565	0.113	0.556	0.584
	SVM	0.213	**0.803**	0.210	0.020	0.508	0.504
	XGBoost	0.534	0.603	0.574	0.138	0.569	0.593

Bold values means the highest values of that column in the tables.

**FIGURE 3:**
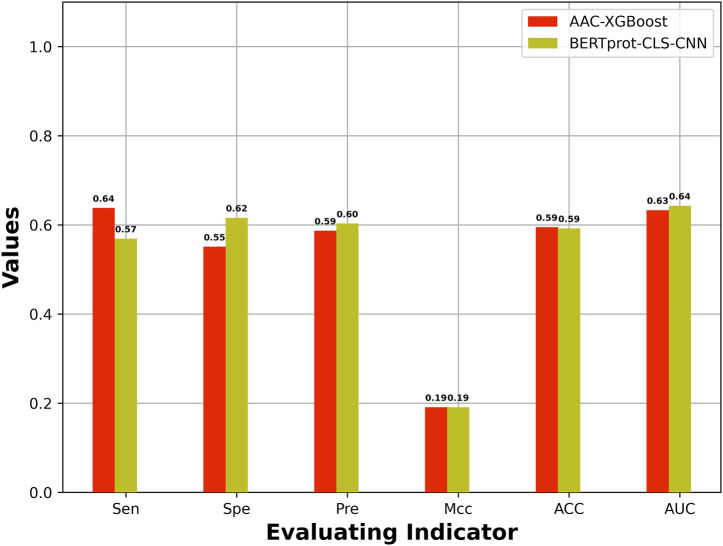
Comparison of performance between the best model based on HCFs and the embeddings extracted from pretrained BERT model.

The best model based on HCFs was compared with the best model based on embeddings of pretrained BERT. Figure 3 shows the best model based on embeddings of BERT-prot, which showed higher AUROC than the best model based on HCFs.

### 3.4 Comparing With Other Existing Methods on the Independent Test Set

**FIGURE 4:**
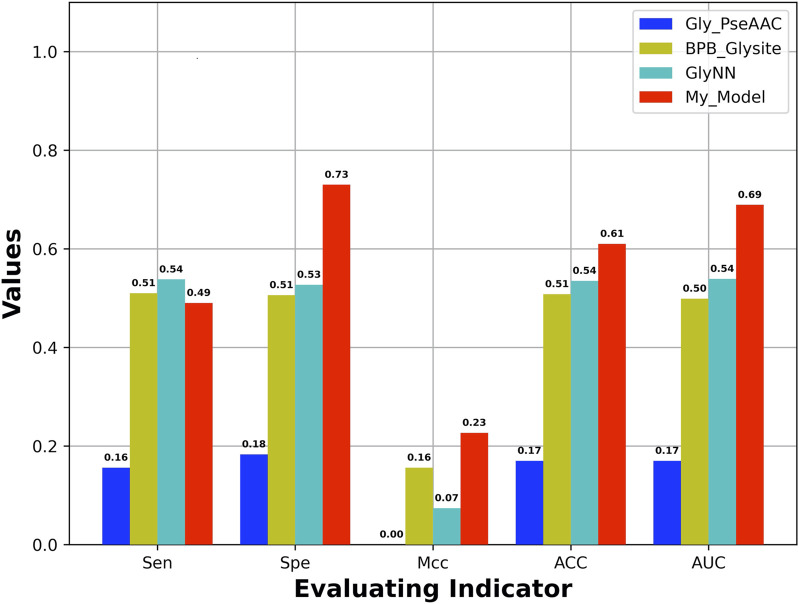
Performance of our model and other existing models based on independent test set.

An independent test set was used to evaluate the generalization of our model, which is obtained from Yu et al.’s work (Yu et al., 2019). In addition, the dataset has also been used to test other four models including GlyNN, Gly-PseAAC, BPB-GlySite, and PredGly. Although about 11 models have been developed (Supplementary Table S1) for predicting Kgly sites, only the four models mentioned previously are available and work well. In addition, the model PredGly has been built with features including KNN encoding whose overfitting nature has been demonstrated in Basith et al.’s work (Basith et al., 2021). Furthermore, the performance could not be recovered when we retrained the model. Thus, we only compared our model with GlyNN, Gly-PseAAC, and BPB-GlySite. As shown in Figure 4, the AUROC, MCC, ACC, and SPE of our model are 0.69, 0.23, 0.61, and 0.73, respectively, which are substantially higher than those of GlyNN, Gly-PseAAc, and BPB-GlySite. Our results indicate that our model is better than other existing predictors, which implies that the features extracted from NLP pretrained models could be useful for predicting protein posttranslational modification sites.

### 3.5 Web Implementation

For the easy use of our model, we deployed a web server at http://bert-kgly.zhulab.org.cn/ *.* The users can carry out the prediction as follows:

First, the input of the server can be protein sequences in text or a FASTA file that contains the query protein sequences. Then, by clicking the “submit” button, a unique task ID would be assigned to the job. To obtain the results, the users can provide their email addresses on the webpage. When the job was done, the results would be sent to the users by email.

## 4 Discussions

In this study, the embeddings of three different pretrained BERT models were extracted to build our models. Our results indicated that the embeddings obtained from BERT-prot which is based on 556,603 protein sequences are more effective than the embeddings extracted from the other two BERT models, although the other two BERT models were pretrained on larger datasets. Generally speaking, the model parameters and the size of the dataset for pretraining are positively related to the representability of the embeddings. Another factor, the domain-specific data have also been reported to be proportionally related to the representability. In our study, one possible reason is that the dataset obtained from UniProt (Swiss-Prot) may be more specific than the dataset obtained from Pfam because the data from Swiss-Prot are from manually curated protein sequences.

**FIGURE 5:**
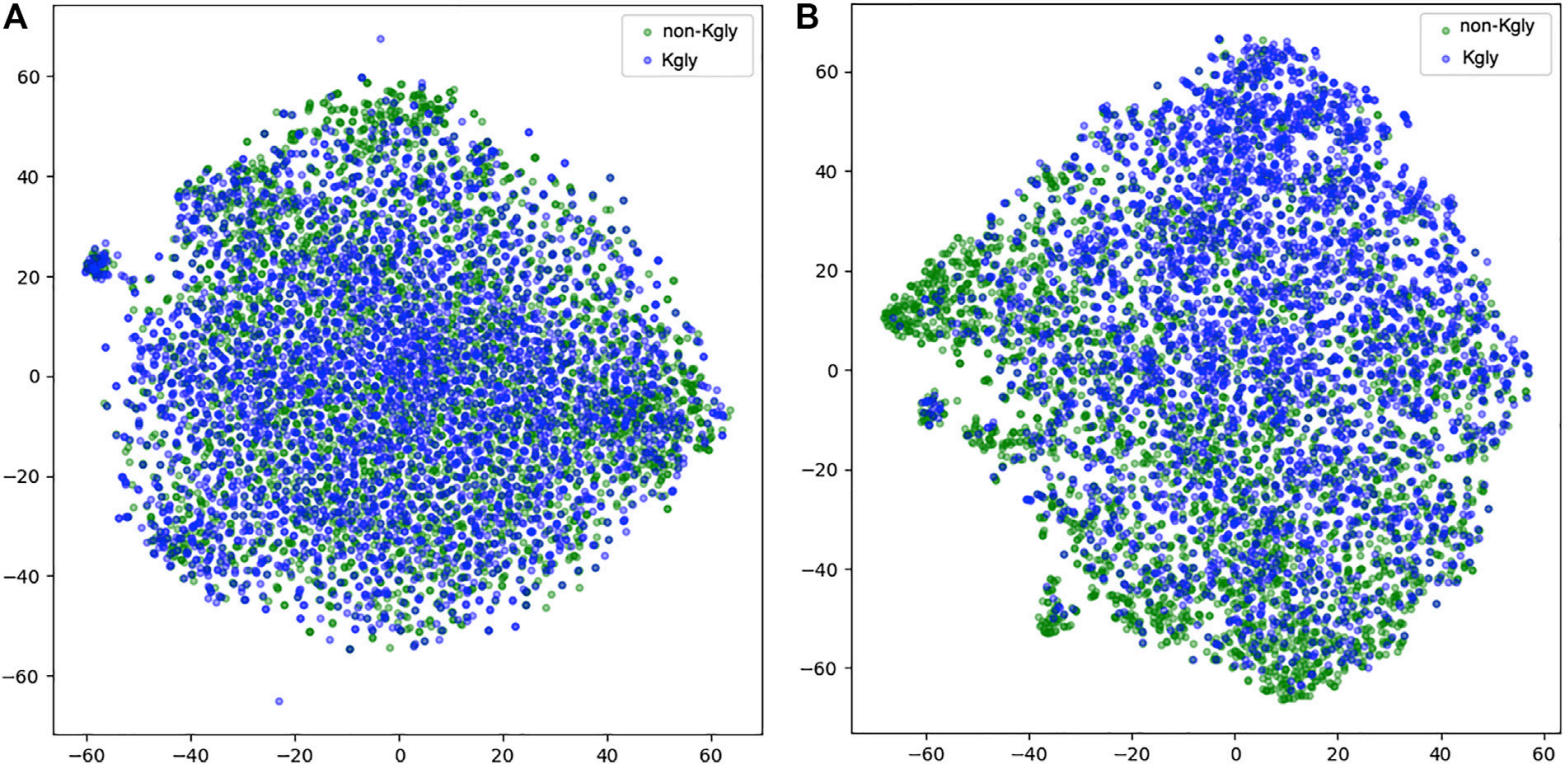
*t*-SNE illustration of the embeddings extracted from BERT and the features transformed by 1D CNN. **(A)** Embeddings extracted from BERT-prot. **(B)** Features transformed by 1D CNN.

To inspect the effectiveness of our 1D-CNN network, we compared the features extracted from BERT-prot and the features transformed by the 1D-CNN network. We used t-SNE to project the features into the two-dimensional space (Figure 5). For the features extracted from BERT-prot for token “CLS,” although there are some clusters for positive or negative samples, overall, the positive and negative samples are tangled together (Figure 5A). However, for the features transformed by 1D CNN, Figure 5B shows that negative samples (green points) are concentrated at the bottom left, while positive samples (blue points) are concentrated at the top right. Thus, we demonstrated that the informative feature representation from input sequences can be learned by the pretrained BERT model and the downstream 1D CNN network.

Considering the information of all residues of the protein segments, we have built the model based on the embeddings of all residues of the whole segment, and the corresponding cross-validation AUROC is 0.646, which is similar to the model based on the embedding of “CLS” (0.643). Additional results showed that the model based on the embeddings of all residues of the whole sequences had worse generalization on the independent test set with an AUROC of 0.624, which is smaller than that of our model based on the embeddings of “CLS.”

To investigate the complementarity between BERT embeddings and HCFs, we combined the embeddings of BERT with the AAC feature, which is the best handcrafted feature in this study. The two features were concatenated as the input of our deep networks. The cross-validation results showed that the corresponding AUROC is 0.6427, which is similar to the highest value (0.643) based only on the embeddings of BERT.

Our model was built and evaluated on balanced datasets; however, the reality is that the negative samples are more than positive samples. So, we have constructed an imbalanced independent test to evaluate our model, which contains 200 positive samples and 1,000 negative samples. We used our model to do prediction on the imbalanced test set and obtained an AUROC of 0.64 for the imbalanced test set. The imbalanced dataset was also tested on other models. Based on the predictive results of Gly-PseAAC and BPB-Glysite, the AUROCs for the two models were calculated which are 0.53 and 0.51, respectively. The results indicated that our model was superior to the two models on the imbalanced test set. Note that the results predicted by the web server of GlyNN could not be displayed normally.

Many studies (Bao et al., 2019a; Bao et al., 2019b; Bao et al. 2021) have been conducted to predict the modifications of lysines. Our results indicated that the embeddings extracted from BERT could be effective features for building the models.

## 5 Summary

In this study, we developed a new method, BERT-Kgly, to predict Kgly sites of proteins by extracting features from a pretrained protein language BERT model. Recently, NLP pretrained models have been transferred to analyze and tackle sequence information of biological macromolecules. Different pretrained protein language BERT models have been built based on different sizes of protein sequences. We adopted two protein language BERT models and one natural language BERT model to extract features from peptide segments. Our results demonstrate the features extracted from BERT-prot are more informative than the other two BERT models. Three different downstream deep networks were used to build our models; it turned out that the model based on 1D CNN was superior to the models based on other two networks. Our model was compared with the models built on HCFs and traditional machine learning algorithms, which indicated that our BERT-Kgly model outperformed these models. Thus, we demonstrate the effectiveness of features extracted from the pretrained protein BERT model and the downstream deep learning networks. In comparison to the independent test set, we also showed that our model was superior to other state-of-the-art models.

## Data Availability

Publicly available datasets were analyzed in this study. These data can be found here: https://github.com/yinboliu-git/Gly-ML-BERT-DL.
