# Supplementary material for: BERT-Kgly: A Bidirectional Encoder Representations From Transformers (BERT)-Based Model for Predicting Lysine Glycation Site for Homo sapiens
Source: Front Bioinform. 2022 Feb 18;2:834153. doi: 10.3389/fbinf.2022.834153 (PMC9580886; doi:10.3389/fbinf.2022.834153)
Supplement: Supplementary file 1 [file DataSheet1.docx]

**BERT-Kgly: A Bidirectional Encoder Representations from Transformers (BERT)-based Model for Predicting Lysine Glycation Site for Homo sapiens**

Yinbo Liu^1#^, Yufeng Liu^1#^, Gangao Wang^1^, Yinchu Cheng^1^, Shoudong Bi^1*^ and Xiaolei Zhu^1*^

^1^ School of Sciences, Anhui Agricultural University, Anhui, China, 230036

**Supplementary Materials**

**Details of BERT**

BERT (bidirectional encoder representations from transformers) model was developed by Devlin et al. [1], which has achieved new state-of-the-art results on 11 natural language processing (NLP) tasks. The architecture of BERT is a multi-layer bidirectional Transformer encoder, which jointly conditions on both left and right context using the attention mechanism in all encoder layers and processes all words in the sentence in parallel. The network structure of all the encoder layers are the same which mainly consisted of two sub-layers: the multi-head self-attention layer and the feed-forward neural network layer. Besides, a residual connection is added on each of the sub-layer, thus, the output of each sublayer is LayerNorm(x+Sublayer(x)). When a sentence is inputted to BERT model, each word was encoded by three embeddings: token embeddings, segment embeddings and position embeddings. Then, we can obtain context-dependent features from different encoder layers of the model.

**References**

1. Devlin J, Chang MW, Lee K, Toutanova K: **BERT: Pre-training of Deep Bidirectional Transformers for Language Understanding**. *Proceedings of the 2019 Conference of the North American Chapter of the Association for Computational Linguistics: Human Language Technologies* 2019, **1**: 4171-4186.

2. Yao Y, Zhao X, Ning Q, Zhou J: **ABC-Gly: Identifying Protein Lysine Glycation Sites with Artificial Bee Colony Algorithm**. *Current Proteomics* 2021, **18**(1):18-26.

3. Ju Z, Sun JH, Li YJ, Wang L: **Predicting lysine glycation sites using bi-profile bayes feature extraction**. *Comput Biol Chem* 2017, **71**:98-103.

4. Khanum S, Ashraf MA, Karim A, Shoaib B, Alswaitti M: **Gly-LysPred: Identification of Lysine Glycation Sites in Protein Using Position Relative Features and Statistical Moments Via Chou's 5 Step Rule**. *Cmc -Tech Science Press-* 2020, **66**(2):2165-2181.

5. Johansen MB, Kiemer L, Brunak S: **Analysis and prediction of mammalian protein glycation**. *Glycobiology* 2006, **16**(9):844-853.

6. Zhao XW, Zhao XS, Bao LL, Zhang YG, Dai JY, Yin MH: **Glypre: In Silico Prediction of Protein Glycation Sites by Fusing Multiple Features and Support Vector Machine**. *Molecules* 2017, **22**(11):15.

7. Xu Y, Li L, Ding J, Wu LY, Mai GQ, Zhou FF: **Gly-PseAAC: Identifying protein lysine glycation through sequences**. *Gene* 2017, **602**:1-7.

8. Islam MM, Saha S, Rahman MM, Shatabda S, Farid DM, Dehzangi A: **iProtGly-SS: Identifying protein glycation sites using sequence and structure based features**. *Proteins* 2018, **86**(7):777-789.

9. Yang Y, Wang H, Li W, Wang X, Wei S, Liu Y, Xu Y: **Prediction and analysis of multiple protein lysine modified sites based on conditional wasserstein generative adversarial networks**. *BMC Bioinformatics* 2021, **22**(1).

10. Yu JL, Shi SP, Zhang F, Chen GD, Cao M: **PredGly: predicting lysine glycation sites for Homo sapiens based on XGboost feature optimization**. *Bioinformatics* 2019, **35**(16):2749-2756.

11. Liu Y, Gu WX, Zhang WY, Wang JN: **Predict and Analyze Protein Glycation Sites with the mRMR and IFS Methods**. *Biomed Res Int* 2015, **2015**:6.

12. Chen Z, Liu X, Li F, Li C, Marquez-Lago T, Leier A, Akutsu T, Webb GI, Xu D, Smith AI *et al*: **Large-scale comparative assessment of computational predictors for lysine post-translational modification sites**. *Brief Bioinform* 2019, **20**(6):2267-2290.

13. Reddy HM, Sharma A, Dehzangi A, Shigemizu D, Chandra AA, Tsunoda T: **GlyStruct: glycation prediction using structural properties of amino acid residues**. *BMC Bioinformatics* 2019, **19**(Suppl 13):547.

**Supplementary Tables**

**Table S1.**A detailed summary of the reviewed tools for Kgly sites prediction.

| **Predictors** | **Data**  **source** | **Datasets ^a^** | **Features** | **Feature selection** | **Classifiers** | **Web servers** | **ref** |
| --- | --- | --- | --- | --- | --- | --- | --- |
| ABC-Gly | CPLM  PLMD3.0 | 260/1100 | PSAAP,SS, CKSAAP | BABC | SVM | - | [2] |
| BPB_GlySite | CPLM | 223/446 | Bi-Profile Bayes | - | SVM | <http://123.206.31.171/>BPB_GlySite/ | [3] |
| Gly-LysPred | UniProt | 1287/1300 | Statistical Moments, PRIM, RPRIM, Frequency Matrix Determination, AAPIV, RAAPIV | - | RF | - | [4] |
| GlyNN | UniProt | 89/126 | The distribution of the relative position | - | ANN | - | [5] |
| Glypre | CPLM UniProt GlycateBase PLMD3.0 | 336/546 | The position scoring function, SS, AAindex, CKSAAP | mRMR GFS | SVM | - | [6] |
| Gly-PseAAC | CPLM | 223/446 | PSAAP | - | SVM | <http://app.aporc.org/>Gly-PseAAC/ | [7] |
| iProtGly-SS | CPLM literatures | 394/689 | AAC, SS Motifs residue physical chemical properties | IFGS | SVM | http://brl.uiu.ac.bd/ | [8] |
| multiLyGAN | CPLM2.0 | 1399/- | AAindex, CKSAAP, PWM Reduced Alphabet Fold Amyloid, BE, PC‑PseAAC,SC‑PseAAC, ASA, SS, BTA, HSE, CN | PCC | RF | - | [9] |
| PredGly | PLMD | 3969/82270 | AAC, PWAA, CKSAAP, DBPB, EBGW, KNN | XGBoost | SVM |  | [10] |
| preGly | GlyNN | 89/126 | AAOF, CKSAAP, AAindex | mRMR IFS | SVM | <http://202.198.129.220>:8080/GlycationPre | [11] |
| MUscADEL | PhosphoSitePlus | 2432/4729 | Word embedding | - | BiLSTM-RNN | http://muscadel.erc.monash.edu/ | [12] |
| GlyStruct | CPLM | 235/1518 | Predicted structure properties of residues | -- | SVM |  | [13] |

^a^ The numbers represent the numbers of positive samples and negative samples.

**Table S2.** The ranges of different hyperparameters used in grid search for the three deep networks.

| **Learning_rate** | **Num_train_epochs** | **Train_batch_size** |
| --- | --- | --- |
| [2e-4,2e-5,2e-6] | [2,4,8,16,32] | [32,64,128] |

**Table S3.** By using embedding of token ‘CLS’ as features, the best hyperparameter combinations for the three deep networks based on AUROC values obtained from the grid search.

| **Pre-trained BERT models** | **Predictor** | **Learning_rate** | **epochs** | **batch_size** |
| --- | --- | --- | --- | --- |
| BERT-Base | 1DCNN | 2.00E-05 | 32 | 32 |
|  | BiLSTM | 2.00E-05 | 32 | 64 |
|  | 1DCNN+BiLSTM | 2.00E-05 | 32 | 64 |
| BERT-prot | 1DCNN | 2.00E-05 | 8 | 32 |
|  | BiLSTM | 2.00E-04 | 16 | 32 |
|  | 1DCNN+BiLSTM | 2.00E-04 | 8 | 32 |
| TAPE | 1DCNN | 2.00E-04 | 4 | 128 |
|  | BiLSTM | 2.00E-05 | 16 | 64 |
|  | 1DCNN+BiLSTM | 2.00E-05 | 2 | 32 |

**Table S4.** By using embedding of token ‘K’ as features, the best hyperparameter combinations for the three deep networks based on AUROC values obtained from the grid search.

| **Pre-trained BERT models** | **Predictor** | **Learning_rate** | **epochs** | **batch_size** |
| --- | --- | --- | --- | --- |
| BERT-Base | 1DCNN | 2.00E-04 | 16 | 128 |
|  | BiLSTM | 2.00E-04 | 16 | 128 |
|  | 1DCNN+BiLSTM | 2.00E-04 | 16 | 128 |
| BERT-prot | 1DCNN | 2.00E-05 | 8 | 64 |
|  | BiLSTM | 2.00E-04 | 4 | 32 |
|  | 1DCNN+BiLSTM | 2.00E-04 | 4 | 32 |
| TAPE | 1DCNN | 2.00E-04 | 16 | 64 |
|  | BiLSTM | 2.00E-05 | 16 | 64 |
|  | 1DCNN+BiLSTM | 2.00E-05 | 16 | 32 |

**Table S5.** By using average embeddings of the peptide segments as features, the best hyperparameter combinations for the three deep networks based on AUROC values obtained from the grid search.

| **Pre-trained BERT models** | **Predictor** | **Learning_rate** | **epochs** | **batch_size** |
| --- | --- | --- | --- | --- |
| BERT-Base | 1DCNN | 2.00E-04 | 16 | 128 |
|  | BiLSTM | 2.00E-04 | 16 | 128 |
|  | 1DCNN+BiLSTM | 2.00E-04 | 16 | 128 |
| BERT-prot | 1DCNN | 2.00E-05 | 8 | 64 |
|  | BiLSTM | 2.00E-04 | 16 | 64 |
|  | 1DCNN+BiLSTM | 2.00E-04 | 8 | 64 |
| TAPE | 1DCNN | 2.00E-06 | 8 | 32 |
|  | BiLSTM | 2.00E-05 | 4 | 64 |
|  | 1DCNN+BiLSTM | 2.00E-05 | 16 | 128 |

**Table S6.** The ranges of different hyperparameters used in grid search for traditional learning classifiers.

| **Classifiers** | **C** | **Gamma** | **N_estimators** | **Max_depth** | **Learning_rate** | **p** |
| --- | --- | --- | --- | --- | --- | --- |
| SVM | [0.5,1,2,4] | [1e-7,1e-6,1e-5,1e-4,  1e-3,1e-2,1e-1] |  |  |  |  |
| RF |  |  | [1600,1800,2000,2200] | [2,4,6,8] |  |  |
| XGBoost |  |  | [1600,1800,2000,2200] | [2,4,6,8] | [0.005,0.01,0.02,0.04] |  |
| KNN |  |  | [2,4,6,8] |  |  | [1,2,3,4] |

**Table S7.** The best hyperparameter combinations of different classifiers for different features based on AUROC values obtained from the grid search.

| **Features** | **classifiers** | **Optimal hyperparameters** |
| --- | --- | --- |
| AAC | Knn | {'n_neighbors': 6, 'p': 3} |
|  | RF | {'max_depth': 8, 'n_estimators': 1800} |
|  | SVM | {'C': 4, 'gamma': 0.0625, 'kernel': 'rbf'} |
|  | XGBoost | {'learning_rate': 0.005, 'max_depth': 4, 'n_estimators': 1800} |
| DBPB | Knn | {'n_neighbors': 6, 'p': 1} |
|  | RF | {'max_depth': 8, 'n_estimators': 2000} |
|  | SVM | {'C': 0.5, 'gamma': 0.015625, 'kernel': 'rbf'} |
|  | XGBoost | {'learning_rate': 0.01, 'max_depth': 8, 'n_estimators': 2200} |
| EBGW | Knn | {'n_neighbors': 6, 'p': 4} |
|  | RF | {'max_depth': 4, 'n_estimators': 2000} |
|  | SVM | {'C': 4, 'gamma': 0.0625, 'kernel': 'rbf'} |
|  | XGBoost | {'learning_rate': 0.005, 'max_depth': 2, 'n_estimators': 2000} |
| KNN | Knn | {'n_neighbors': 6, 'p': 1} |
|  | RF | {'max_depth': 2, 'n_estimators': 2000} |
|  | SVM | {'C': 4, 'gamma': 0.0625, 'kernel': 'rbf'} |
|  | XGBoost | {'learning_rate': 0.005, 'max_depth': 2, 'n_estimators': 2200} |
| CKSAAP | Knn | {'n_neighbors': 6, 'p': 1} |
|  | RF | {'max_depth': 8, 'n_estimators': 1800} |
|  | SVM | {'C': 0.5, 'gamma': 0.0078125, 'kernel': 'rbf'} |
|  | XGBoost | {'learning_rate': 0.005, 'max_depth': 8, 'n_estimators': 1800} |
| PWAA | Knn | {'n_neighbors': 6, 'p': 3} |
|  | RF | {'max_depth': 8, 'n_estimators': 2000} |
|  | SVM | {'C': 4, 'gamma': 0.0625, 'kernel': 'rbf'} |
|  | XGBoost | {'learning_rate': 0.005, 'max_depth': 2, 'n_estimators': 2200} |

**Supplementary Figures**


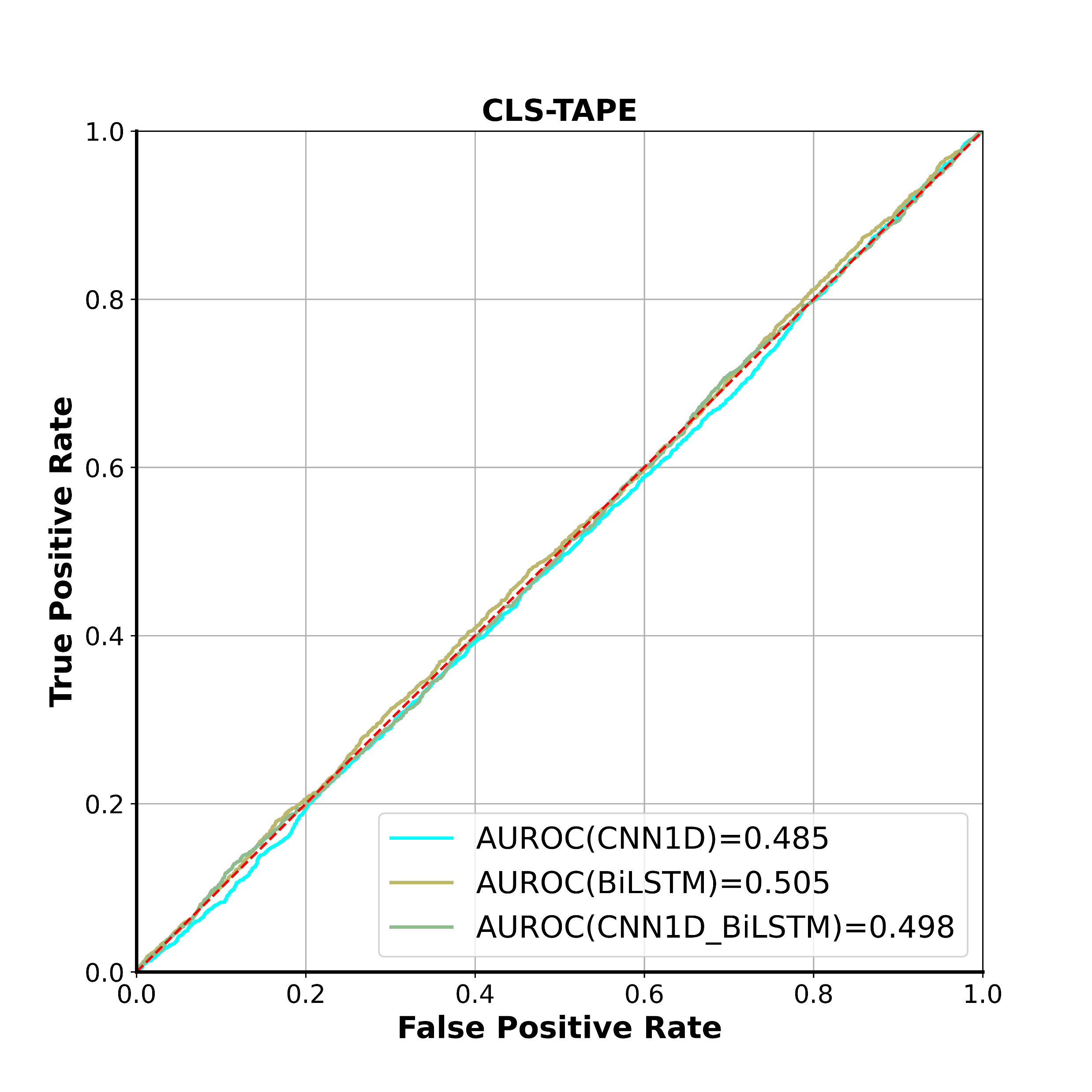

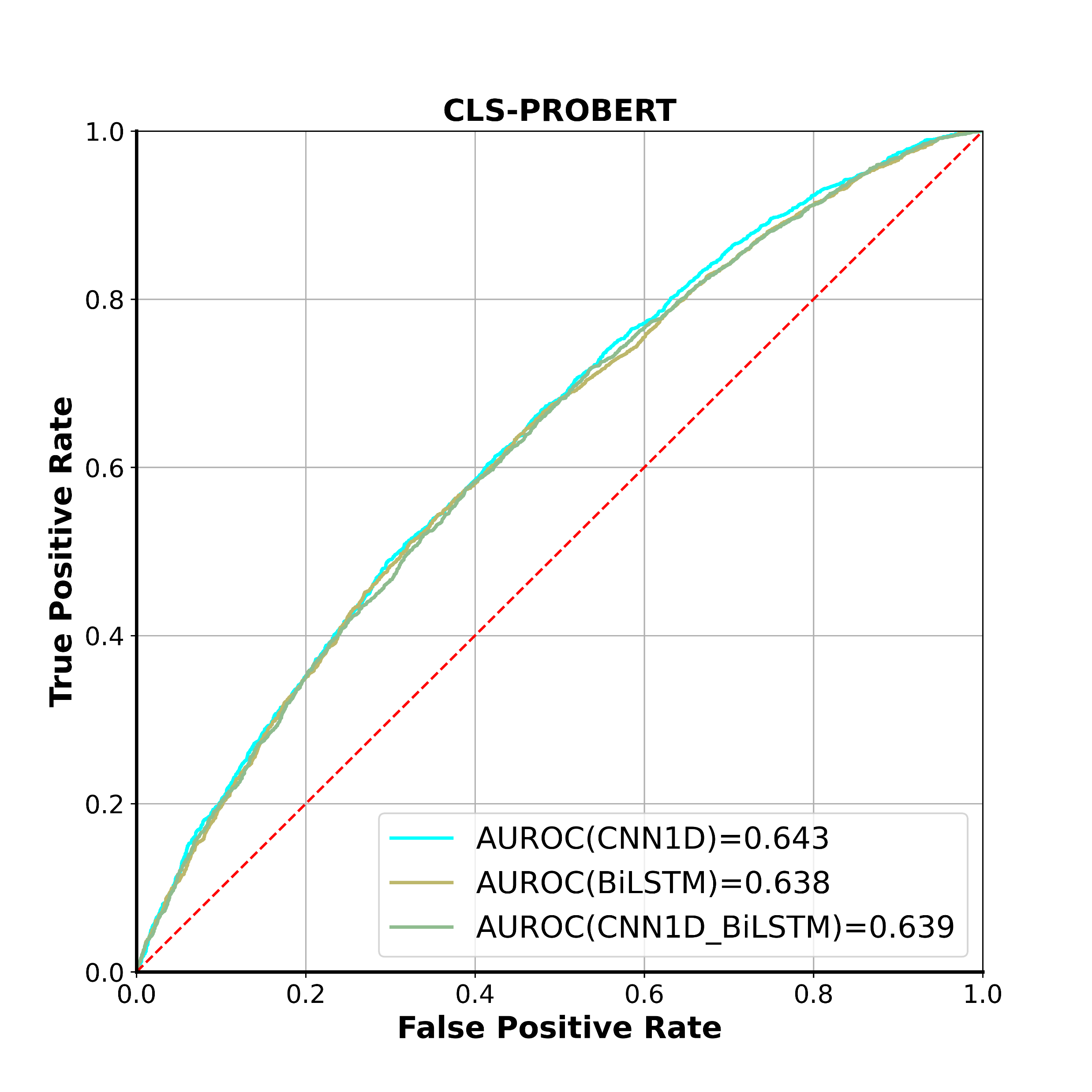


b

c


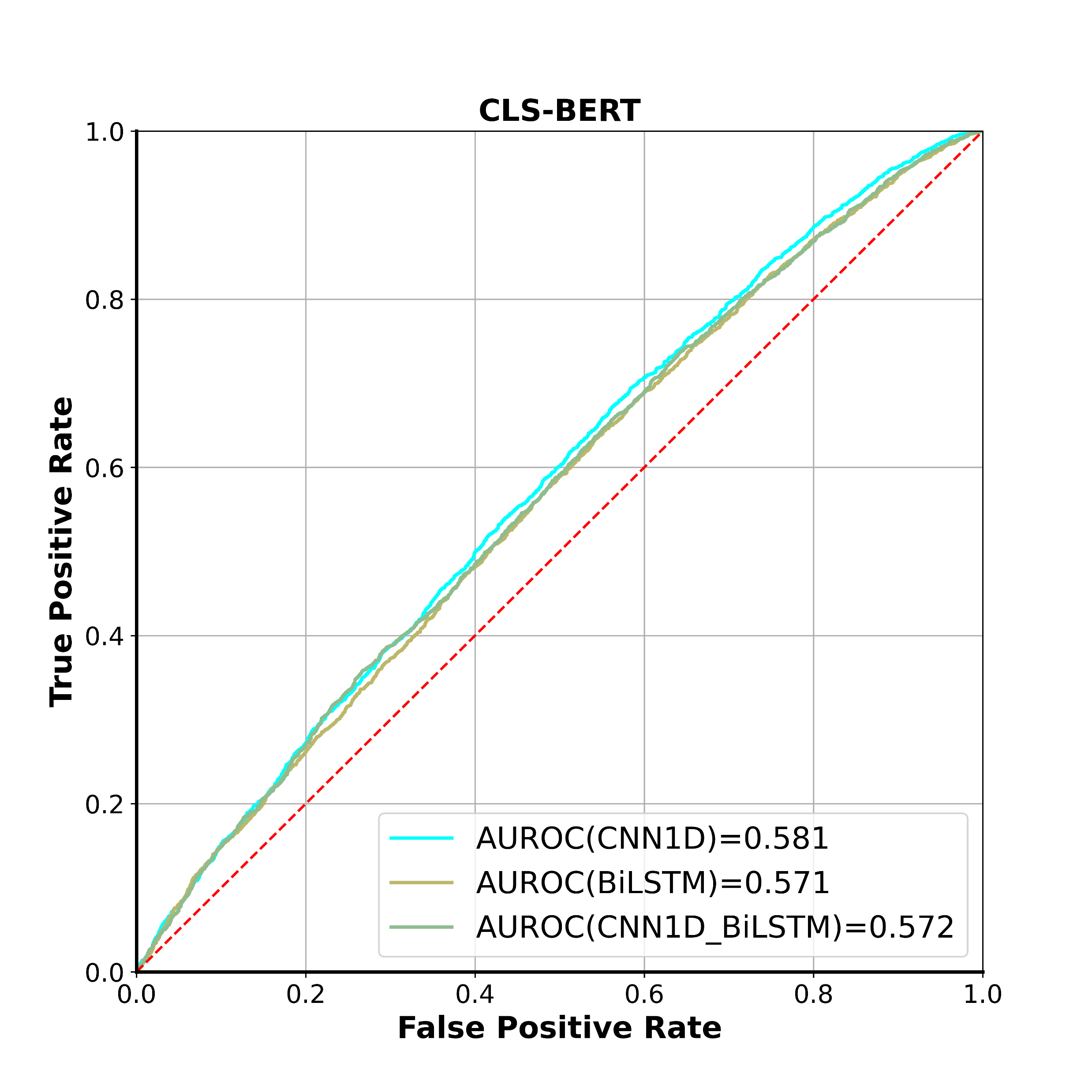

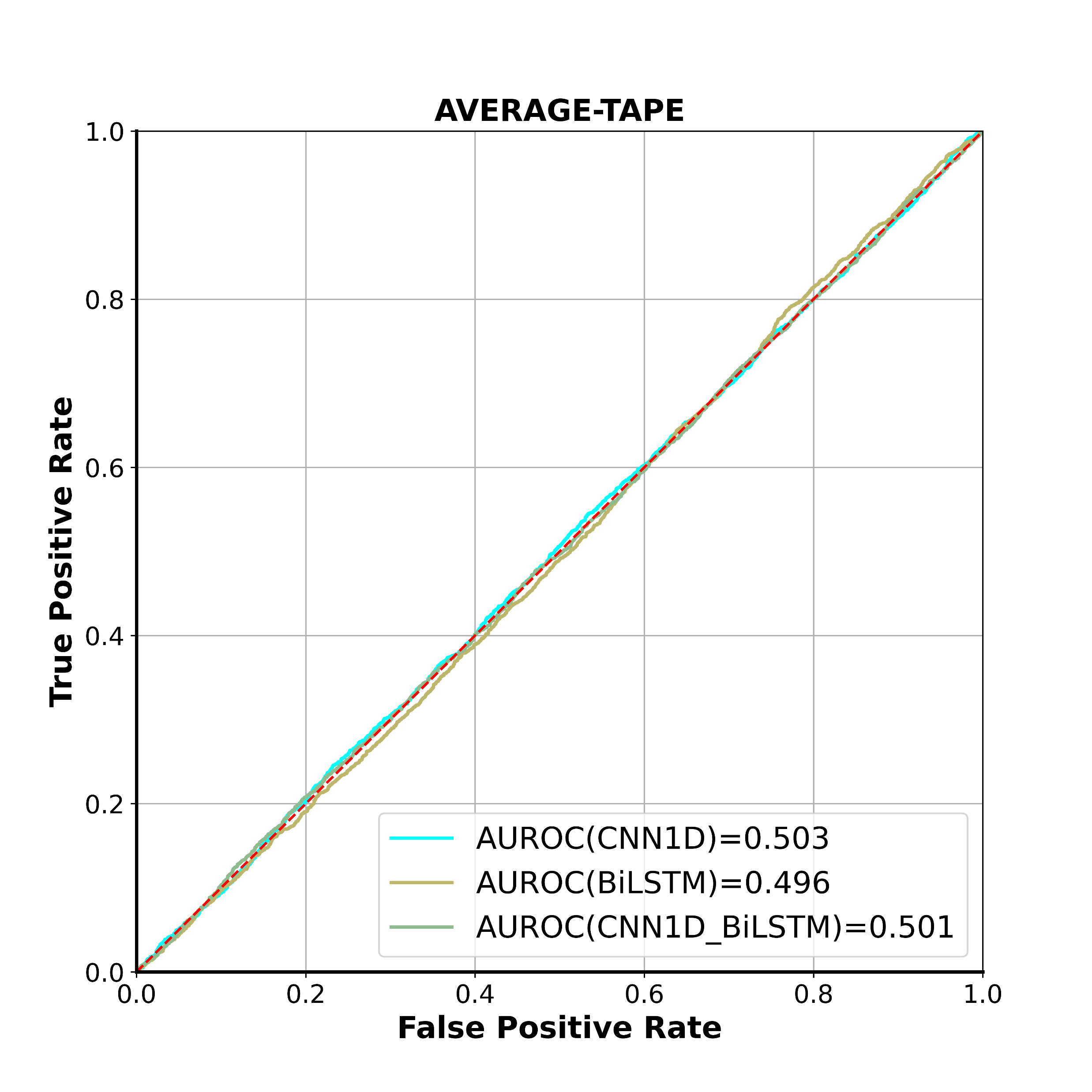

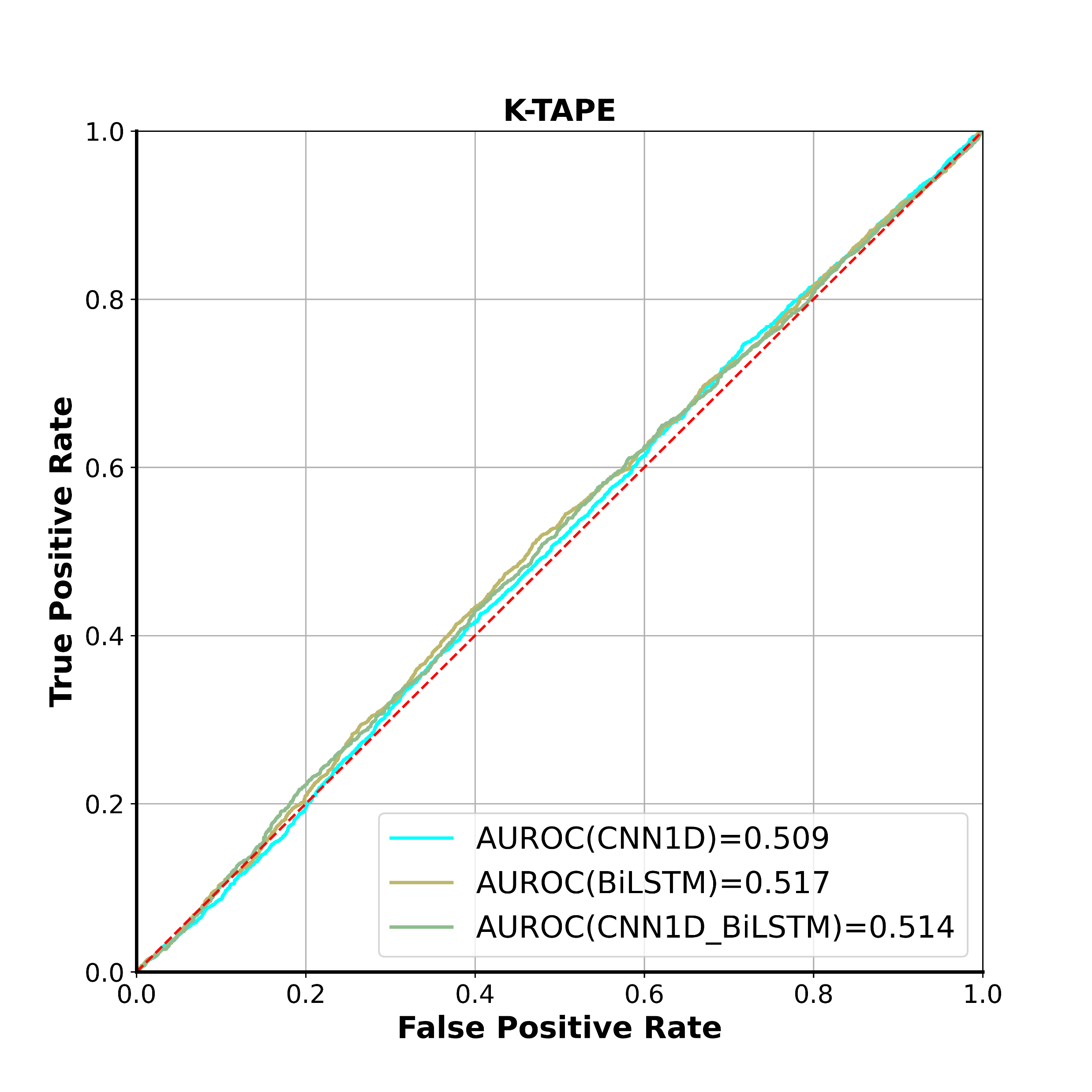

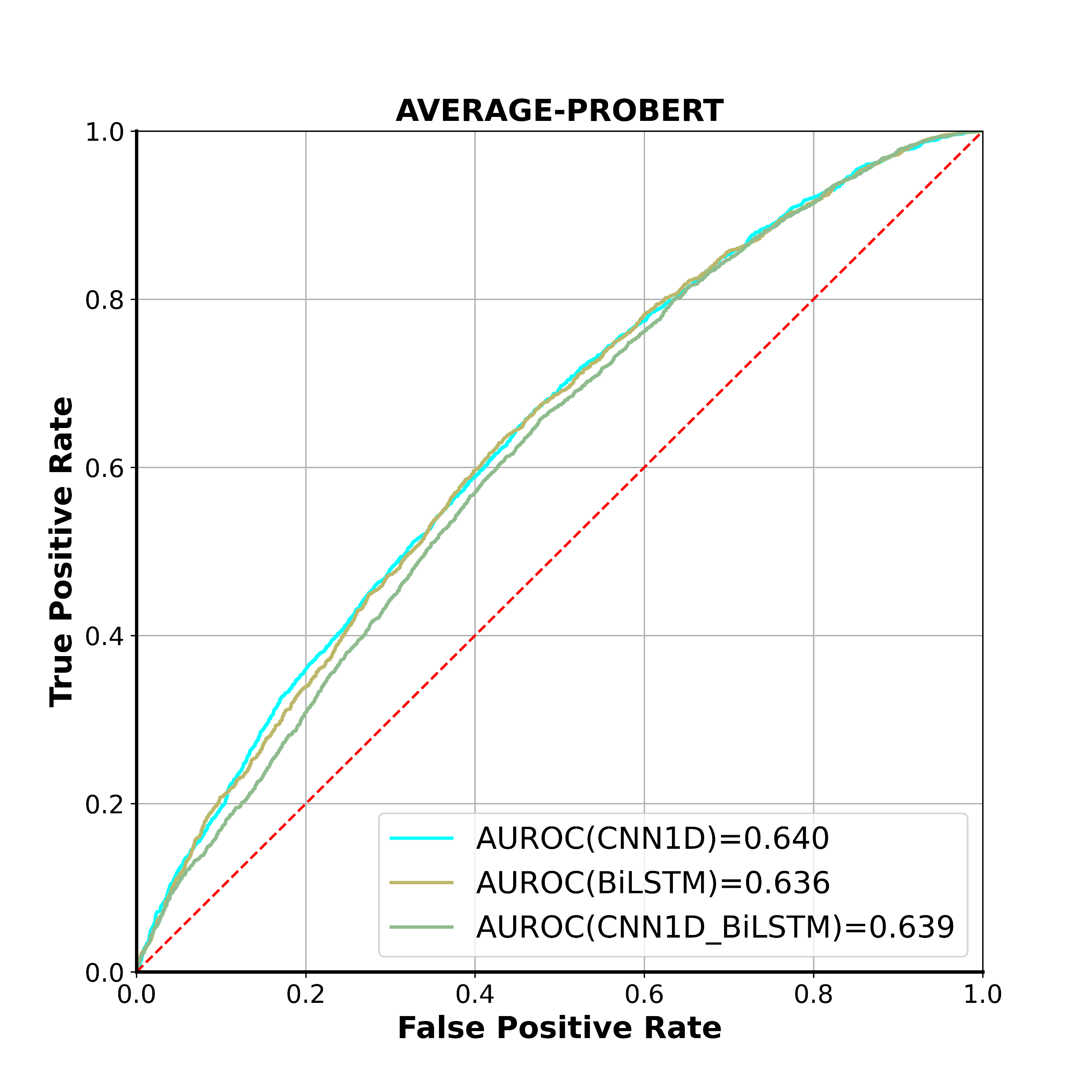

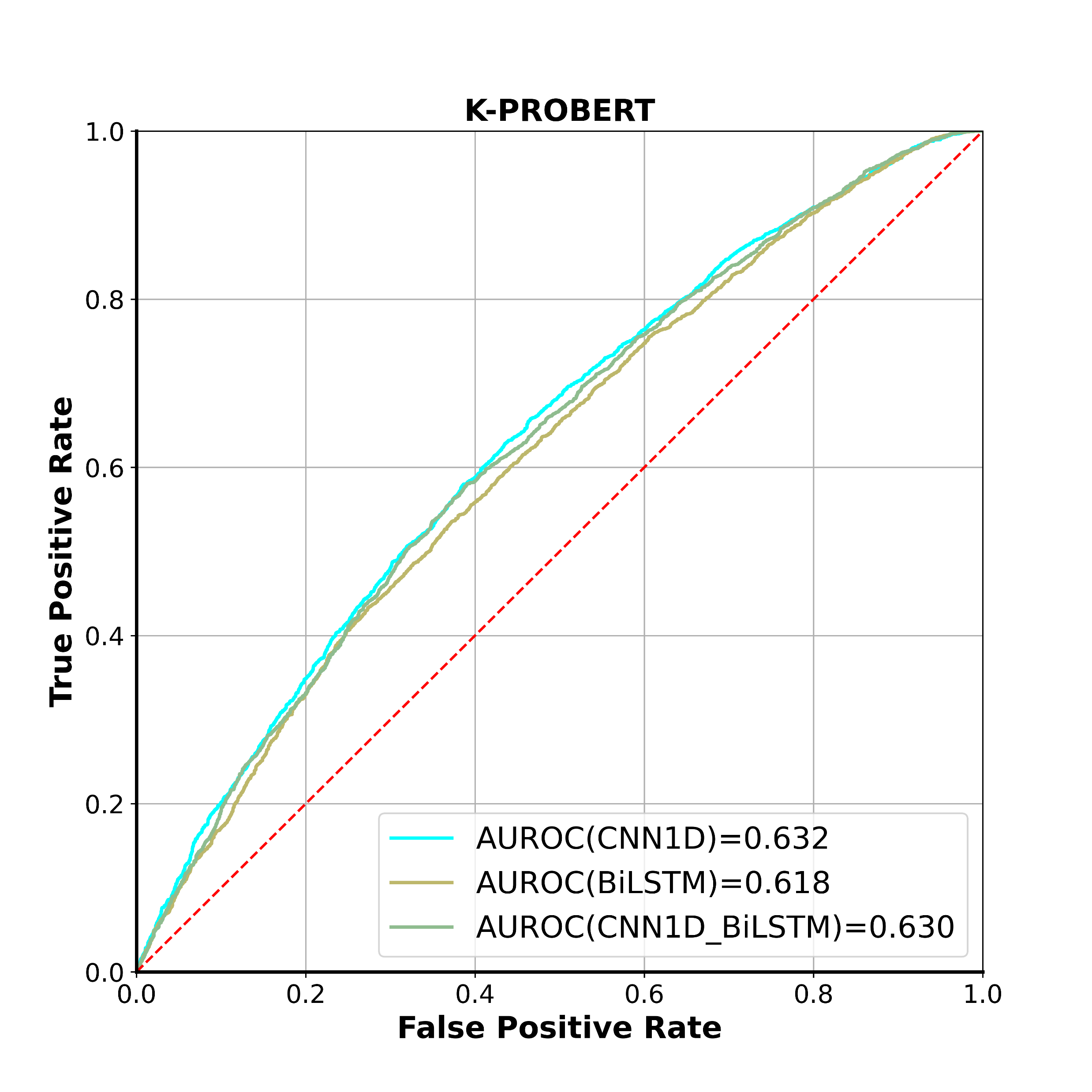

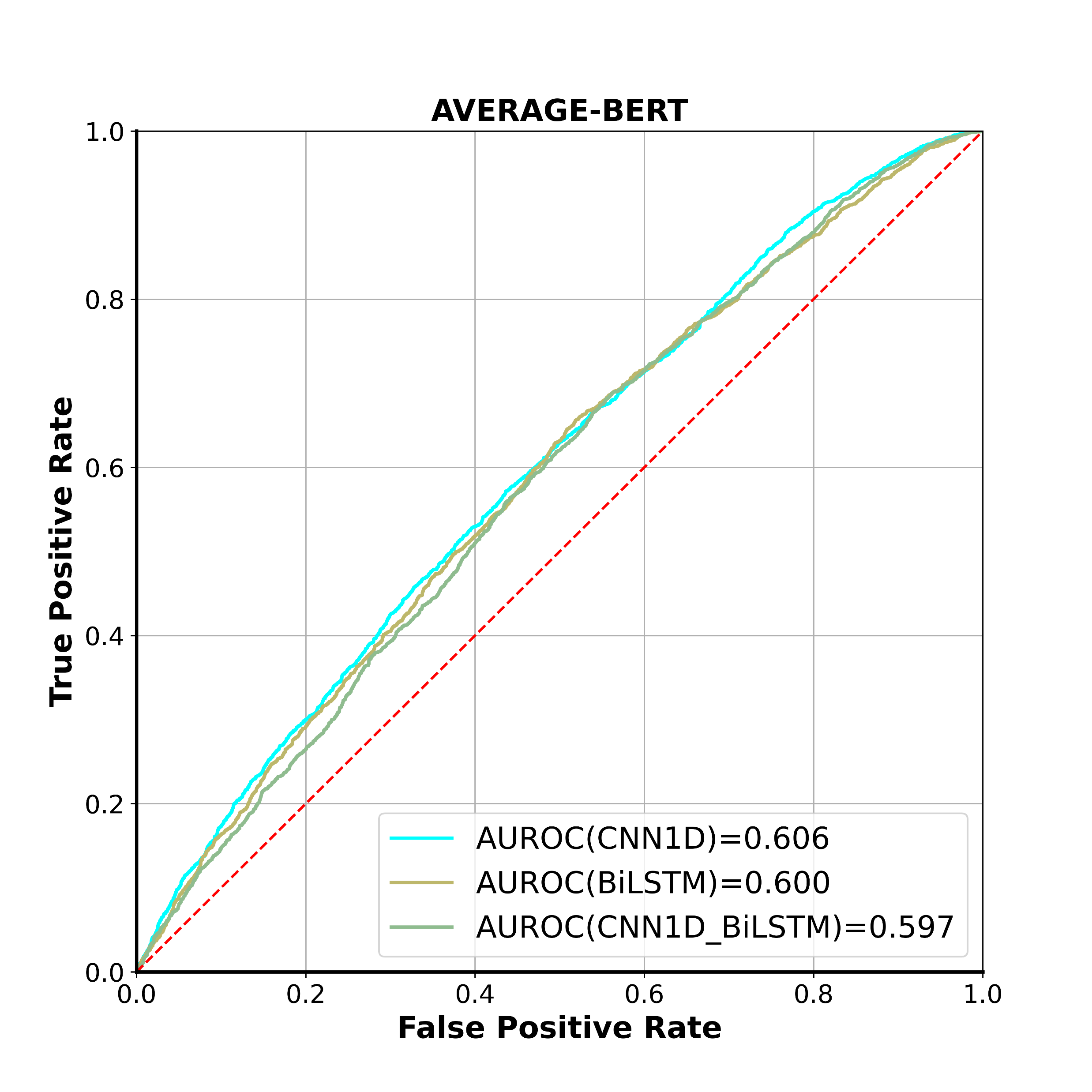

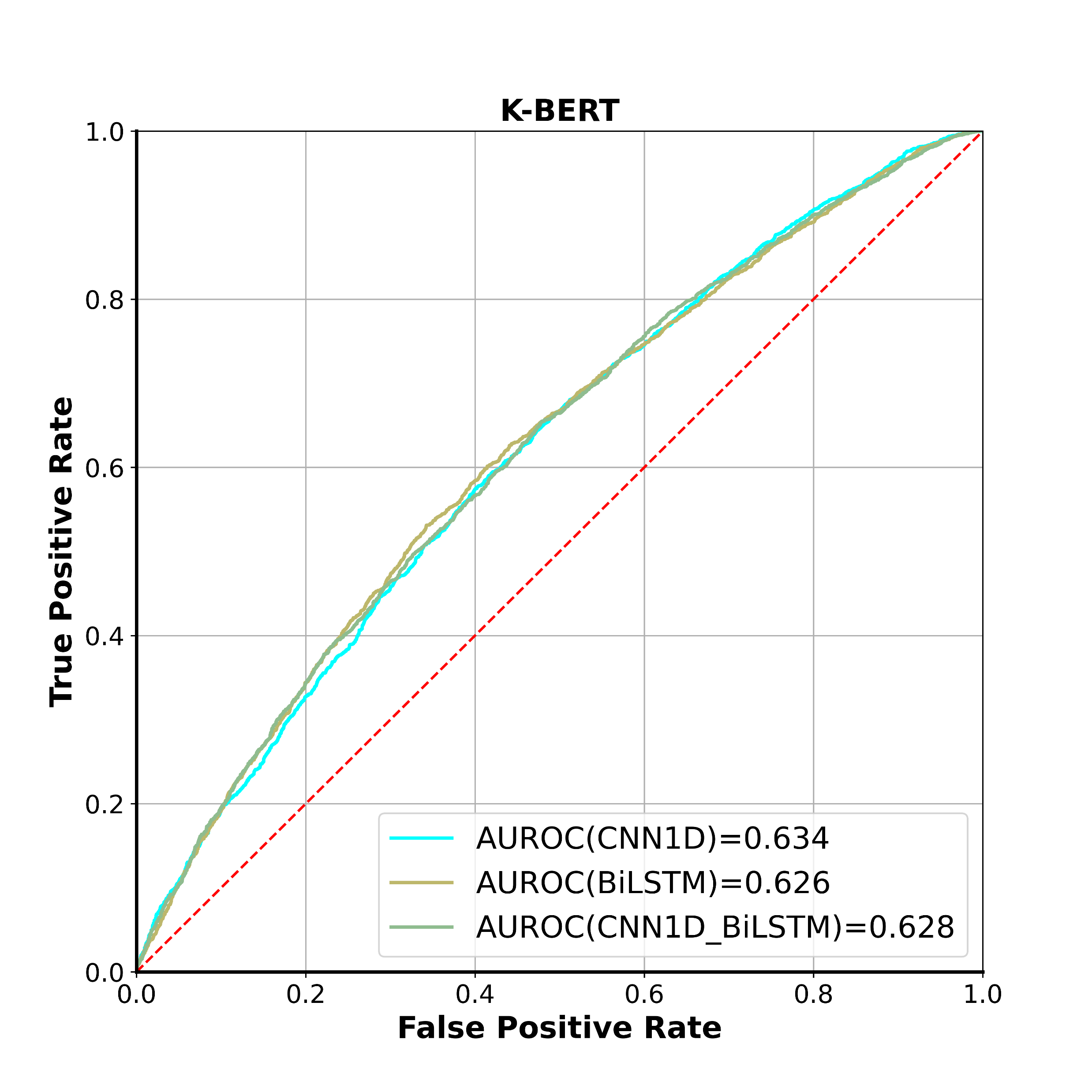


a

Figure S1. Cross-validation ROC curves for models based on embeddings extracted from BERT models.


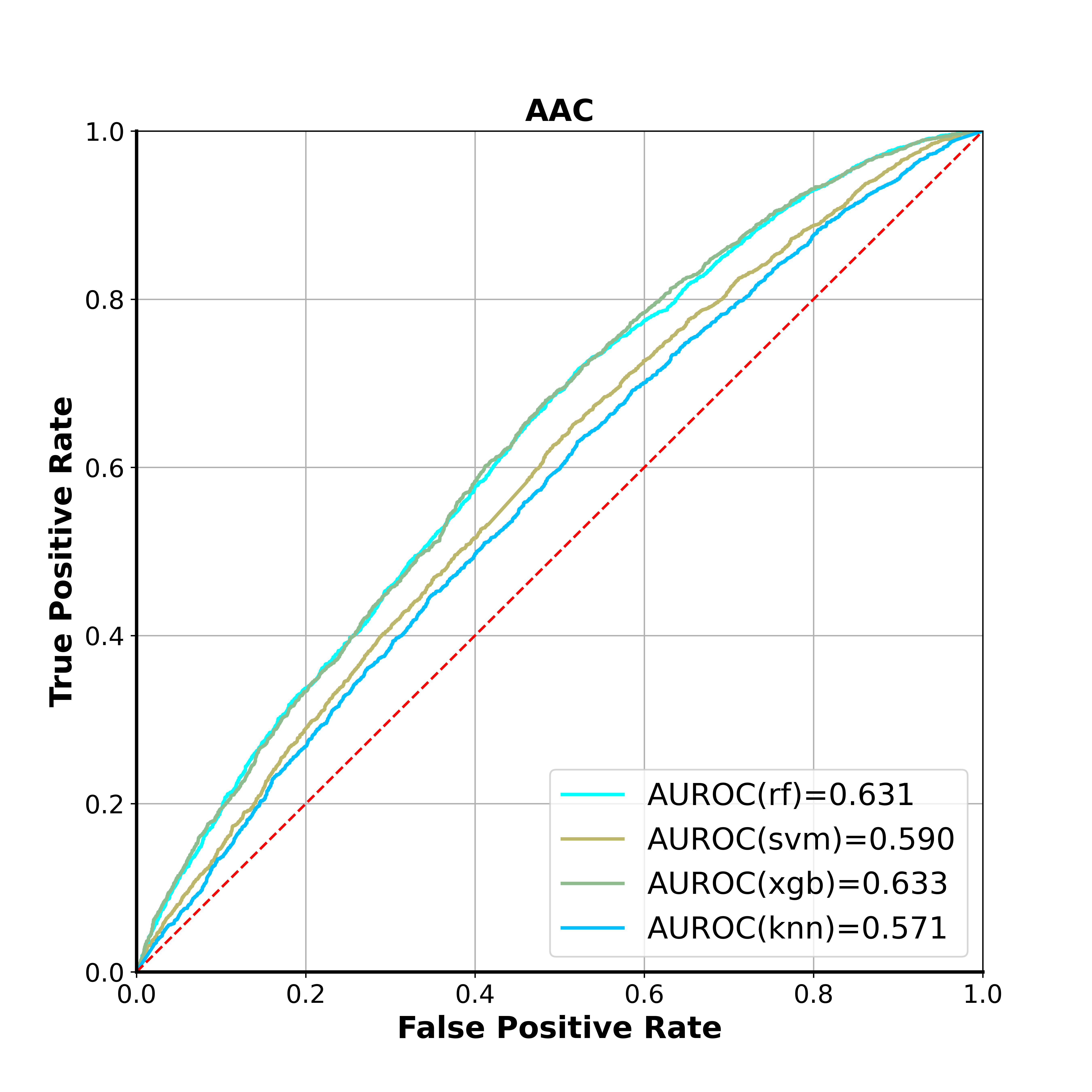

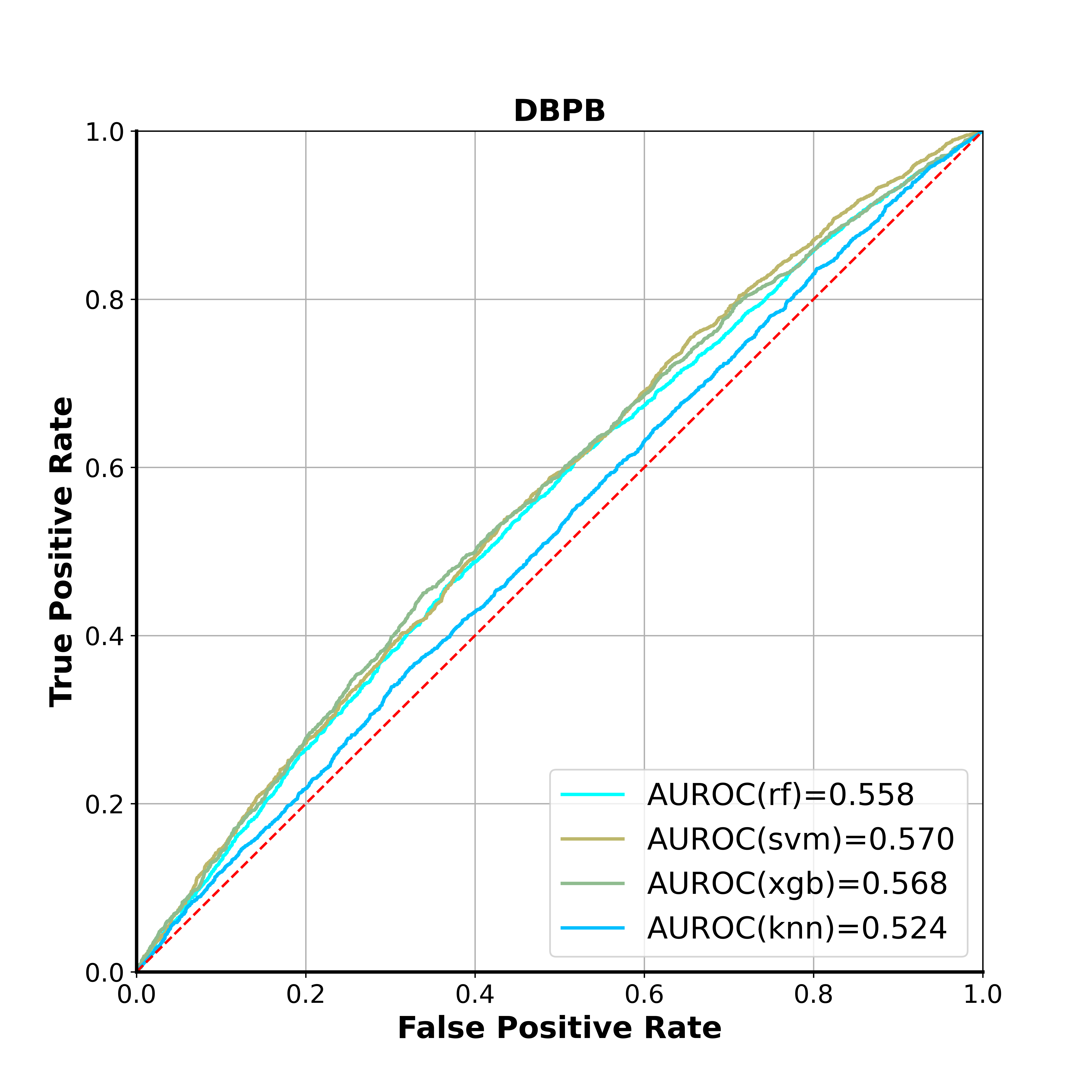

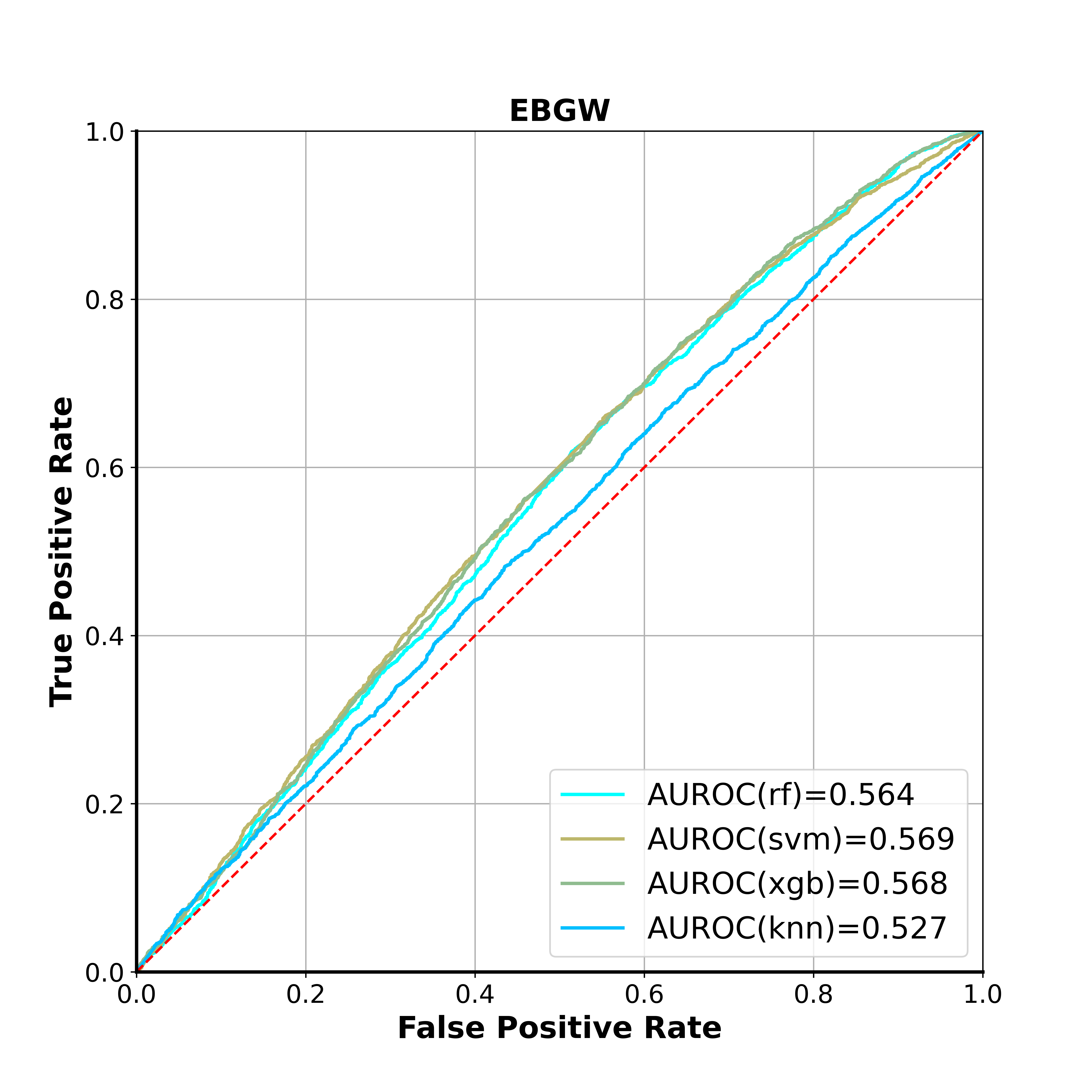

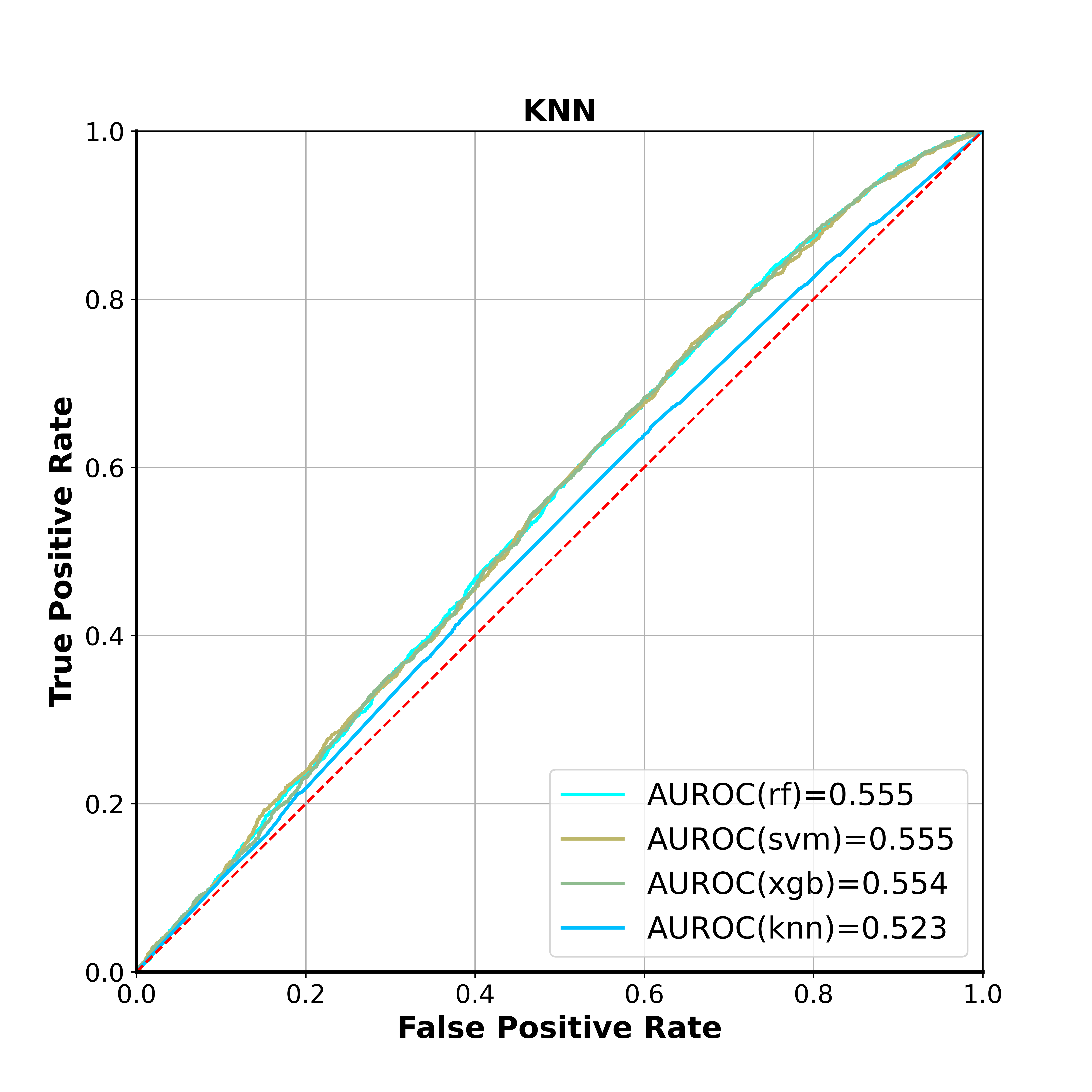

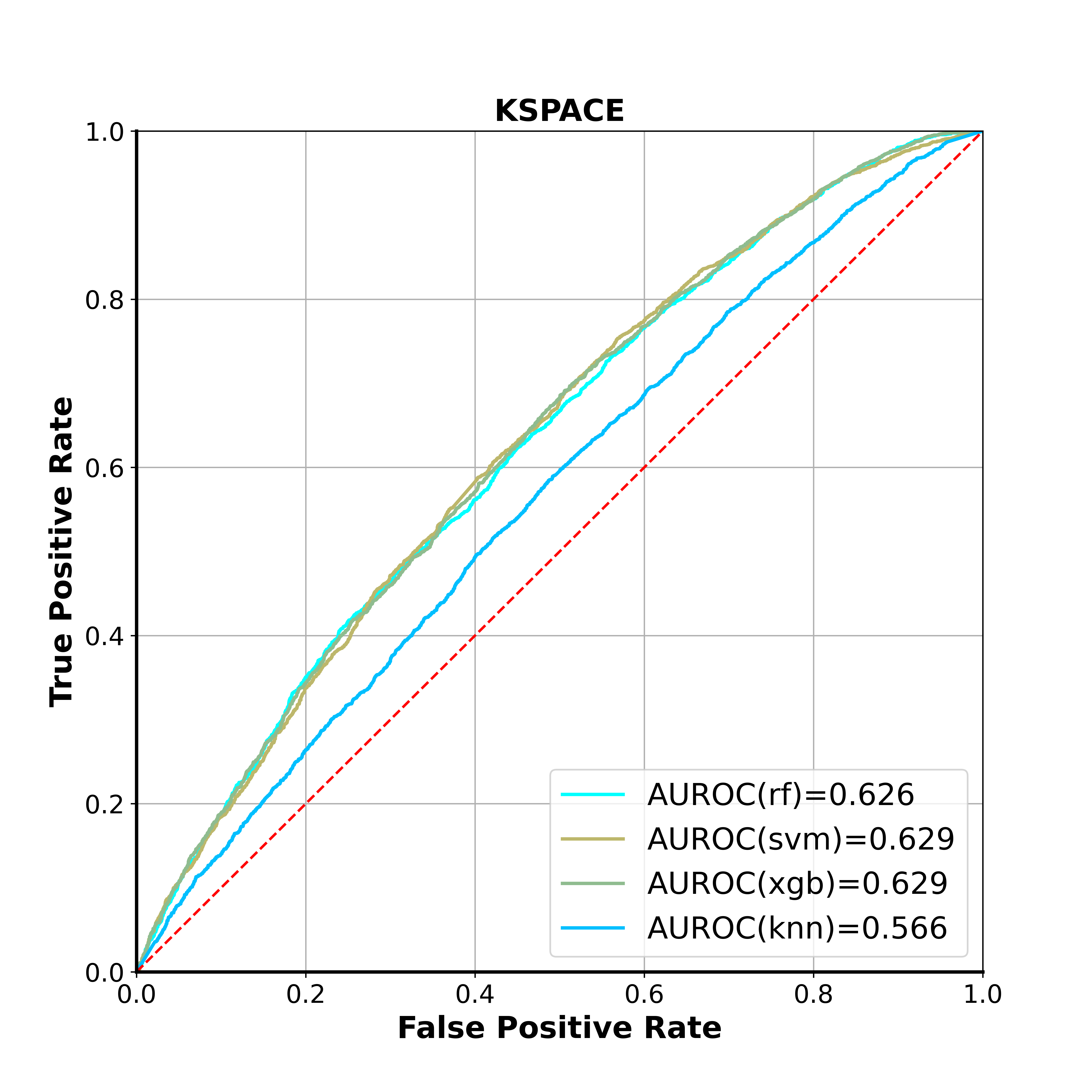

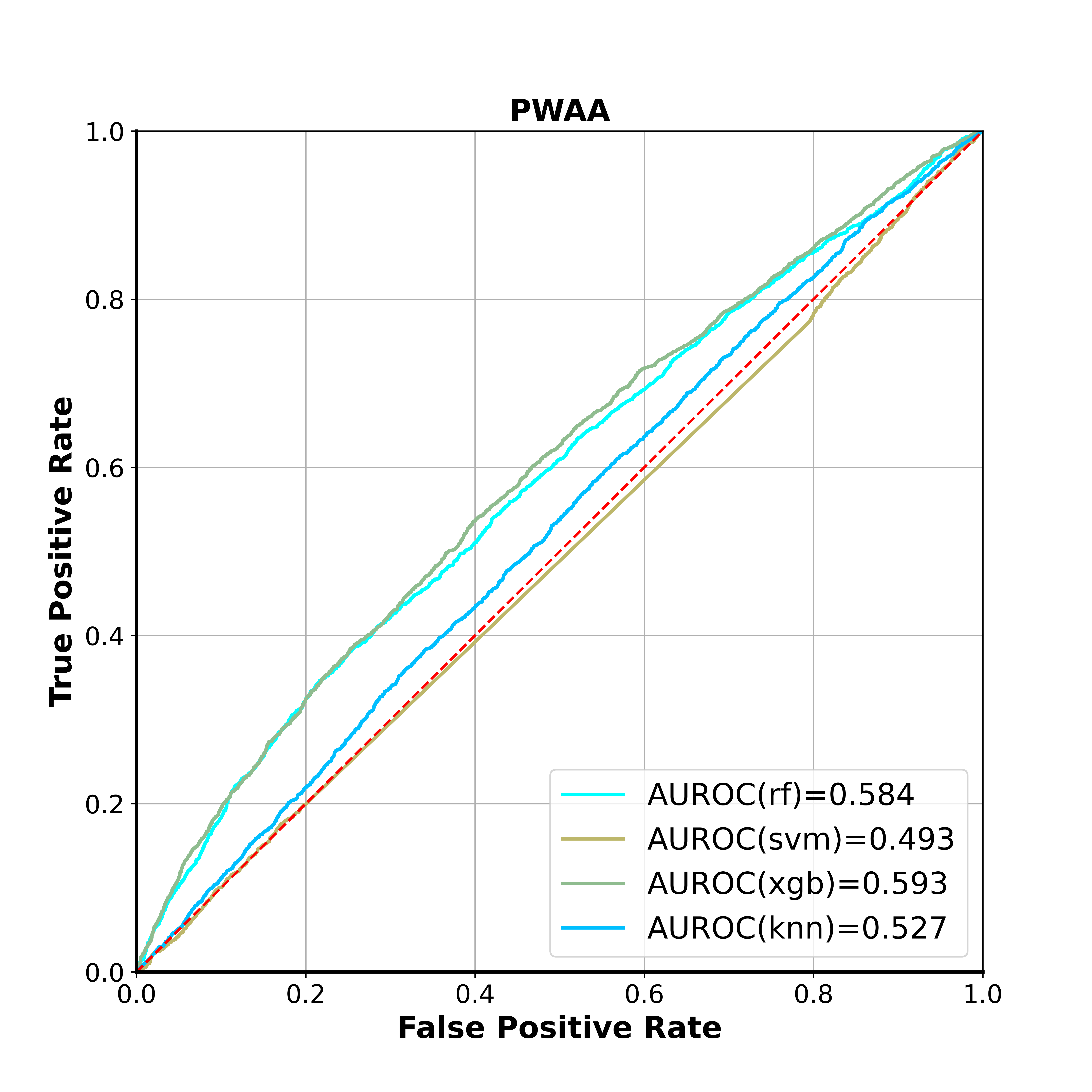


Figure S2. Cross-validation ROC curves for models based on HCF features.
